# Multi-modal mechanisms of the metastasis suppressor, NDRG1: Inhibition of WNT/β-catenin signaling by stabilization of protein kinase Cα

**DOI:** 10.1016/j.jbc.2024.107417

**Published:** 2024-05-28

**Authors:** Mahan Gholam Azad, Mohammed Hussaini, Tiffany M. Russell, Vera Richardson, Busra Kaya, Mahendiran Dharmasivam, Des R. Richardson

**Affiliations:** 1 Centre for Cancer Cell Biology and Drug Discovery, Griffith University, Brisbane, Queensland, Australia; 2 Department of Pathology and Biological Responses, Nagoya University Graduate School of Medicine, Nagoya, Japan

**Keywords:** N-myc downstream regulated gene-1, protein kinase Cα, β-catenin, metastasis, signaling, pancreatic cancer

## Abstract

The metastasis suppressor, N-myc downstream regulated gene-1 (NDRG1), inhibits pro-oncogenic signaling in pancreatic cancer (PC). This investigation dissected a novel mechanism induced by NDRG1 on WNT/β-catenin signaling in multiple PC cell types. NDRG1 overexpression decreased β-catenin and downregulated glycogen synthase kinase-3β (GSK-3β) protein levels and its activation. However, β-catenin phosphorylation at Ser33, Ser37, and Thr41 are classically induced by GSK-3β was significantly increased after NDRG1 overexpression, suggesting a GSK-3β-independent mechanism. Intriguingly, NDRG1 overexpression upregulated protein kinase Cα (PKCα), with *PKCα* silencing preventing β-catenin phosphorylation at Ser33, Ser37, and Thr41, and decreasing β-catenin expression. Further, NDRG1 and PKCα were demonstrated to associate, with PKCα stabilization occurring after NDRG1 overexpression. PKCα half-life increased from 1.5 ± 0.8 h (3) in control cells to 11.0 ± 2.5 h (3) after NDRG1 overexpression. Thus, NDRG1 overexpression leads to the association of NDRG1 with PKCα and PKCα stabilization, resulting in β-catenin phosphorylation at Ser33, Ser37, and Thr41. The association between PKCα, NDRG1, and β-catenin was identified, with the formation of a potential metabolon that promotes the latter β-catenin phosphorylation. This anti-oncogenic activity of NDRG1 was multi-modal, with the above mechanism accompanied by the downregulation of the nucleo-cytoplasmic shuttling protein, p21-activated kinase 4 (PAK4), which is involved in β-catenin nuclear translocation, inhibition of AKT phosphorylation (Ser473), and decreased β-catenin phosphorylation at Ser552 that suppresses its transcriptional activity. These mechanisms of NDRG1 activity are important to dissect to understand the marked anti-cancer efficacy of NDRG1-inducing thiosemicarbazones that upregulate PKCα and inhibit WNT signaling.

Many studies have demonstrated the anti-oncogenic and anti-metastatic activities of N-myc downstream-regulated gene 1 (NDRG1) in a variety of aggressive solid tumors, including pancreatic cancer, breast cancer, colon cancer, and prostate cancer (1, 2, 3, 4).

The anti-oncogenic and anti-metastatic activities of NDRG1 are mediated by its ability to inhibit a broad range of oncogenic pathways responsible for angiogenesis, tumor growth, and metastasis (5, 6, 7, 8, 9, 10). NDRG1 is composed of 394 amino acids, which includes multiple structural motifs including helix-turn-helix near the *N*-terminus, an inactive α/β hydrolase motif that encompasses a phosphopantetheine attachment site, and a cap-like domain, and three tandem repeats near the *C*-terminus (11, 12).

The oncogenic signaling pathways modulated by NDRG1 include WNT/β-catenin (7, 13), transforming growth factor-β (TGF-β) (9), rho-associated, coiled-coil-containing protein kinase 1/myosin light chain 2 (14), phosphatidylinositol-3-kinase (PI3K)/protein kinase B (AKT) (9, 15), and pathways involving the receptor tyrosine kinases, *for example,* the epidermal growth factor receptor (EGFR), human epidermal growth factor receptor 2 (HER2), c-mesenchymal epithelial transition (c-met), insulin-like growth factor receptor (IGFR), *etc.* (16, 17, 18, 19). The molecular mechanism of action of NDRG1 is mediated *via* its association with other proteins such as the tumor suppressor, mitogen-inducible gene-6 (MIG6), leading to MIG6 stabilization that downregulates EGFR *via* a lysosomal degradation mechanism (17).

WNT/β-catenin signaling is tightly regulated by the destruction complex consisting primarily of axin1, casein kinase 1α (CK1α), adenomatous polyposis coli (APC), and glycogen synthase kinase-3β (GSK-3β) (7, 13). Classically, GSK-3β is involved in β-catenin phosphorylation at Ser33, Ser37, and Thr41 causing its destabilization and degradation by the proteasome (7, 13). The destruction complex is regulated by the binding of WNT ligands to the frizzled and LRP5/6 receptors, with the disheveled protein binding to these receptors (7, 13). The interaction of these receptors with disheveled leads to the dissociation of the destruction complex, resulting in the accumulation of cytoplasmic β-catenin and its nuclear translocation (7, 13). Once β-catenin translocates to the nucleus, it acts as a transcriptional co-activator of transcription factors of the T cell factor and lymphoid enhancer factor-1 family that transactivate oncogenic target genes, such as those encoding c-Myc and cyclin D1 (20, 21).

Aberrant WNT/β-catenin signaling has been demonstrated in many cancers such as pancreatic, colorectal, melanoma, hepatocellular carcinoma, and others (22, 23). Previous studies from our laboratory demonstrated that NDRG1 expression inhibited the TGF-β-induced epithelial-to-mesenchymal transition (EMT) (7). The ability of NDRG1 to inhibit the EMT occurs through (1) maintaining the cell membrane localization of E-cadherin and β-catenin that forms part of the adherens junction complex; (2) inhibiting the expression of the EMT marker, vimentin; and (3) blocking cell migration and invasion of prostate and colon cancer cells (7).

Protein kinase Cα (PKCα) is a member of the protein kinase C family of threonine- and serine-specific protein kinases that require calcium and diacylglycerol for activation (24). The PKCα isotype is ubiquitously expressed and is activated in response to a series of stimuli depending on the effector, and can result in PKCα translocation from the cytoplasm to specialized cellular compartments (24). Due to PKCα being sensitive to multiple stimuli (24), it has been implicated in apoptosis, proliferation, differentiation, inflammation, and motility (24). While PKCα plays a role in a range of pathways, there is increasing evidence that PKCα can regulate WNT/β-catenin signaling (20, 24, 25, 26). In fact, PKCα can associate with, and phosphorylate β-catenin at Ser33, Ser37, and Thr41, causing its degradation and the inhibition of WNT/β-catenin signaling (27).

Pancreatic cancer (PC) is a leading cause of cancer-related death, having the highest mortality-to-incidence rate ratio among all cancers (28, 29). Pharmacological targeting of WNT signaling is a potential therapeutic modality for this belligerent condition (30), with NDRG1 and NDRG1-inducing drugs of the thiosemicarbazone class suppressing the oncogenic WNT pathway and key downstream effectors such as cyclin D1 (13, 31, 32, 33). In fact, novel NDRG1-inducing thiosemicarbazones demonstrate pronounced activity against pancreatic cancer models *in vitro* and *in vivo* (16, 18, 34, 35, 36) and also other tumors (36, 37, 38, 39, 40, 41, 42). These studies underline the importance of understanding the effector roles of NDRG1 and its molecular mechanism of action (for reviews see (5, 43, 44, 45)).

Using several PC cell-types, the current study investigated the antagonistic effect of NDRG1 expression on WNT/β-catenin signaling and demonstrated the inhibition of this pathway by multi-modal mechanisms. These inhibitory activities of NDRG1 included: (1) the identification of a potential metabolon between PKCα, NDRG1, and β-catenin that could promote their catalytic activity. In fact, NDRG1 overexpression results in association of NDRG1 with PKCα and the stabilization of PKCα that phosphorylates β-catenin at Ser33, Ser37, and Thr41 leading to β-catenin degradation; (2) the decreased phosphorylation of β-catenin at Ser552 that suppresses its transcriptional activation (46); and (3) the downregulation of the nucleo-cytoplasmic shuttling protein p21-activated kinase 4 (PAK4), which prevents oncogenic nuclear translocation of β-catenin. Moreover, we report that innovative NDRG1-inducing thiosemicarbazone drugs mimic genetic overexpression of NDRG1 to upregulate PKCα and downregulate β-catenin and its key downstream effector, cyclin D1.

## Results

### NDRG1 overexpression in PC cells increases phosphorylation of β-catenin at Ser33, Ser37, and Thr41 and downregulates the β-catenin nucleo-shuttling protein, PAK4

**Figure 1:**
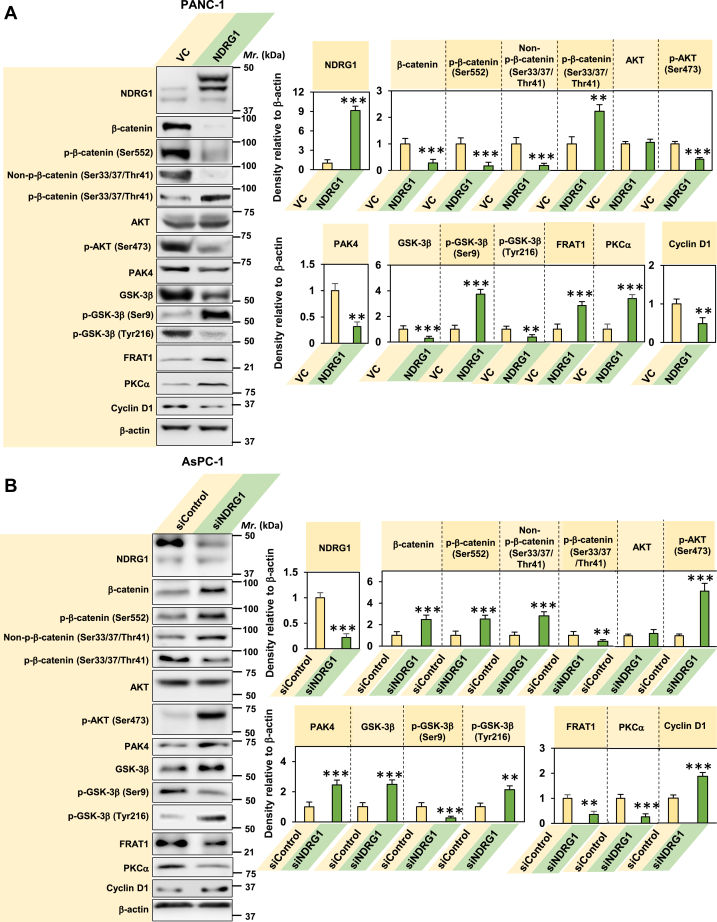
**NDRG1overexpression in PC cells increases phosphorylation of β-catenin at Ser33, Ser37, and Thr41 and downregulates the β-catenin nucleo-shuttling protein, PAK4.***A* and *B*, NDRG1 overexpression decreases activation of WNT/β-catenin signaling in PANC-1 PC cells, while silencing *NDRG1* in AsPC-1 PC cells results in an opposite effect. PANC-1 vector control (VC) and PANC-1 NDRG1 overexpressing cells (NDRG1) were incubated with a control medium at 37 °C for 24 h/37 °C. Total cell protein lysate was then subjected to SDS-PAGE and Western blot analysis. β-actin was used as a protein loading control. *B*, AsPC-1 PC cells were incubated in control medium for 24 h/37 °C. The cells were then transiently transfected with *NDRG1* siRNA or non-targeting negative control siRNA (siControl) for 24 h/37 °C. Total cell lysate was subsequently subjected to SDS–PAGE and Western blot analysis as in (A). Results are presented as the mean ± SD (*n =* 3). Statistical significance is denoted as ∗∗*p* < 0.01 and ∗∗∗*p* < 0.001 comparing VC cells *versus* NDRG1 overexpressing cells.

PC cells display aberrant WNT signaling (22, 23), with previous studies demonstrating that NDRG1 overexpression antagonizes the WNT/β-catenin pathway in colon and prostate cancer cells (13). Since NDRG1 can also decrease nuclear β-catenin levels in PC cells (47), the mechanism of its activity in these tumor cells was important to examine. To investigate the effect of NDRG1 expression on WNT/β-catenin signaling, PANC-1 PC cells were stably transfected with an NDRG1 expression vector compared to their vector control (VC) transfected counterparts (Fig. 1*A*). Western blot analysis was initially performed to examine the effect of NDRG1 overexpression on the total cellular levels of key WNT/β-catenin pathway proteins.

The expression of NDRG1 was significantly (*p* < 0.001) upregulated in NDRG1 overexpressing cells *versus* VC cells (Fig. 1*A*). In VC cells, two endogenous bands for NDRG1 were detected at ∼41 kDa and ∼46 kDa (Fig. 1*A*), which are consistent with the previously reported NDRG1 isoforms that are the result of cleavage, processing, and/or differential phosphorylation (12, 48, 49). The upper (∼46 kDa) NDRG1 band is the key active isoform for metastasis suppression since (1) it is potently upregulated by bespoke NDRG1-inducing thiosemicarbazone drugs that inhibit tumor growth and metastasis (12, 31, 34); and (2) silencing the *NDRG1* top band prevented it acting on downstream effectors (*e.g.*, E-cadherin and β-catenin) that curtail the EMT and metastasis (7). In NDRG1 overexpressing cells, total NDRG1 levels were significantly (*p* < 0.001) greater than that observed in VC cells (Fig. 1*A*). In these latter cells, the flag-tagged transfected NDRG1 was detected at ∼46- and 47-kDa, and the endogenous isoform was observed as a faint band at ∼41 kDa (Fig. 1*A*). The two isoforms of transfected NDRG1 could represent different post-translational modifications of the protein, as observed for endogenous NDRG1 (12, 31, 34). The densitometric analysis presented throughout this study represents the total of all NDRG1 bands detected in each cell-type.

Examining PANC-1 cells, western analysis demonstrated a marked and significant (*p* < 0.001) decrease in total β-catenin levels in the NDRG1 overexpressing cells *versus* the VC (Fig. 1*A*). This was clearly different from studies using prostate and colon cancer cells where NDRG1 expression caused no significant (*p* > 0.05) alteration in total β-catenin (13). The levels of β-catenin phosphorylation at Ser552, which is known for promoting β-catenin transcriptional activity *via* protein kinase B (AKT) (46), was also significantly (*p* < 0.001) decreased upon NDRG1 overexpression relative to the VC (Fig. 1*A*).

In NDRG1 overexpressing PANC-1 cells, levels of non-phosphorylated β-catenin at Ser33, Ser37, and Thr41 decreased significantly (*p* < 0.001) relative to the VC (Fig. 1*A*). Considering this, non-phosphorylated β-catenin (Ser33, Ser37, Thr41) represents β-catenin in its most stable form (50). In contrast, NDRG1 overexpression resulted in a significant (*p* < 0.01) increase in β-catenin phosphorylated at Ser33, Ser37, and Thr41 (Fig. 1*A*), which causes destabilization of this protein and could, at least in part, explain the decreased total β-catenin levels. Therefore, this investigation demonstrates a novel mechanism of β-catenin regulation *via* NDRG1 in PC cells.

As AKT plays an important role in regulating the phosphorylation of β-catenin at Ser552 (46) and GSK-3β at Ser9 (51), its total protein levels and the activating phosphorylation of AKT at Ser473 (52) were also examined. As reported by our laboratory previously using PANC-1 cells (9), total AKT levels were not significantly (*p* > 0.05) affected by NDRG1 overexpression, while its phosphorylation at Ser473 was significantly (*p* < 0.001) decreased *versus* VC cells (Fig. 1*A*). This inactivation of AKT could explain the decreased phosphorylation of β-catenin at Ser552, but not the significant (*p* < 0.001) increase in GSK-3β phosphorylation at Ser9 after NDRG1 overexpression (described below; Fig. 1*A*).

The overexpression of NDRG1 also significantly (*p* < 0.01) downregulated PAK4 (Fig. 1*A*) which is involved in the stability and nuclear translocation of β-catenin (53, 54). Therefore, the ability of NDRG1 overexpression to downregulate PAK4 expression, as well as total β-catenin levels, underlines the anti-oncogenic activity of NDRG1 overexpression in PANC-1 PC cells.

### NDRG1 overexpression induces a GSK-3β-independent mechanism to decrease β-catenin expression that may be mediated by PKCα upregulation

The phosphorylation of β-catenin at Ser33, Ser37, and Thr41, which results in β-catenin degradation, is classically mediated by GSK-3β (55). Interestingly, in PANC-1 PC cells, the total levels of GSK-3β were markedly (*p* < 0.001) decreased upon NDRG1 overexpression *versus* the VC (Fig. 1*A*). Moreover, as described earlier, the inhibitory phosphorylation of GSK-3β at Ser9 (56) was significantly (*p* < 0.001) increased in NDRG1 overexpressing cells (Fig. 1*A*). In contrast, the activating phosphorylation of GSK-3β at Tyr216 (57) was significantly (*p* < 0.01) decreased in NDRG1 overexpressing cells (Fig. 1*A*). The decrease in total GSK-3β and its reduced activation after NDRG1 overexpression in PANC-1 PC cells demonstrates two significant points. First, GSK-3β was probably not responsible for the significant increase in β-catenin phosphorylation at Ser33, Ser37, and Thr41. Second, our study highlights a distinctive difference in the effect of NDRG1 overexpression in PC cells relative to our previous findings (13) using colon and prostate cancer cells where no significant (*p* > 0.05) change in total GSK-3β levels or its activation was observed.

However, similar to our previous investigation examining colon and prostate cancer cells (13), NDRG1 overexpression in PANC-1 cells significantly increased (*p* < 0.001) the expression of the GSK-3β-binding protein, frequently rearranged in advanced T-cell lymphoma (FRAT1), relative to VC cells (Fig. 1*A*). FRAT1 prevents GSK-3β association with the axin1-APC-CK1 destruction complex and the subsequent phosphorylation of β-catenin (58). This effect leads to the accumulation of non-phosphorylated β-catenin after NDRG1 expression in colon and prostate cancer cells (13). Since FRAT1 prevents phosphorylation of β-catenin by GSK-3β (58), these results further suggest the observed ability of NDRG1 overexpression in PC cells to increase β-catenin phosphorylation at Ser33, Ser37, and Thr41 (Fig. 1*A*) is not achieved by GSK-3β. This hypothesis is investigated in detail below.

While FRAT1 levels are increased in PANC-1 cells after NDRG1 overexpression, there was no increase in non-phosphorylated β-catenin (Fig. 1*A*). In fact, a pronounced and significant (*p* < 0.001) decrease in non-phosphorylated β-catenin was observed after NDRG1 overexpression *versus* the VC (Fig. 1*A*). These results indicate the NDRG1-mediated mechanism regulating β-catenin expression in PC cells is distinct from that observed in prostate and colon cancer cells (13).

Since GSK-3β did not appear responsible for the phosphorylation of β-catenin at Ser33, Ser37, and Thr41, it was hypothesized that PKCα could be responsible since it is known to phosphorylate β-catenin at these sites (27). The current studies demonstrated that NDRG1 overexpression significantly (*p* < 0.001) increased total PKCα levels *versus* the VC (Fig. 1*A*), suggesting a potential role of PKCα in β-catenin phosphorylation. The ability of NDRG1 overexpression to decrease total β-catenin expression and increase its phosphorylation at Ser 33, Ser37, and Thr41 should inhibit WNT signaling (59). Considering this, the expression of a key downstream effector of WNT signaling, namely cyclin D1, was significantly (*p* < 0.01) decreased after NDRG1 overexpression *versus* the VC (Fig. 1*A*).

The upregulation of PKCα by the metastasis suppressor, NDRG1, is also of interest since PKCα acts as a tumor suppressor in other cancers because it promotes phosphatase 2A (PP2A)-family-dependent AKT inactivation (60). As such, the increase in PKCα observed after NDRG1 overexpression may lead to decreased p-AKT (Ser473) levels, which then results in decreased β-catenin phosphorylation at Ser552 (Fig. 1*A*). This hypothesis is examined further below in *NDRG1* and *PKCα* silencing studies.

Considering the results above after NDRG1 overexpression in PANC-1 cells, another PC cell-type was also utilized, namely AsPC-1, with *NDRG1* being silenced to examine its effect on WNT β-catenin signaling and PKCα expression (Fig. 1*B*). The AsPC-1 cells were used for silencing as our previous studies demonstrated that it has high endogenous NDRG1 levels enabling effective *NDRG1* silencing (35).

### *NDRG1* silencing causes an opposite response to NDRG1 overexpression: WNT/β-catenin pathway activation, increased GSK-3β activity, and downregulation of PKCα

Examining AsPC-1 PC cells with *NDRG1* silencing, NDRG1 levels were significantly (*p* < 0.001) decreased *versus* the nontargeting negative control siRNA (siControl; Fig. 1*B*). In contrast to NDRG1 overexpression (Fig. 1*A*), after *NDRG1* silencing, total β-catenin levels, the activating phosphorylation of β-catenin at Ser552, and non-phosphorylated-β-catenin (Ser33/37/Thr41) increased significantly (*p <* 0.001) relative to the siControl (Fig. 1*B*). On the other hand, *NDRG1* silencing significantly (*p* < 0.01) decreased p-β-catenin (Ser33/37/Thr41) levels *versus* the siControl (Fig. 1*B*), which was opposite to that observed with NDRG1 overexpression (Fig. 1*A*). In contrast to NDRG1 overexpression (Fig. 1*A*), upon *NDRG1* silencing, PAK4 was significantly (*p* < 0.001) increased *versus* the siControl (Fig. 1*B*) that would promote nuclear translocation of β-catenin (53, 54). Overall, *NDRG1* silencing leads to an opposite response to NDRG1 overexpression, increasing total β-catenin levels potentially by decreasing its phosphorylation at Ser33, Ser37, and Thr41, and promoting its nuclear translocation *via* PAK4.

Opposite to NDRG1 overexpression, *NDRG1* silencing significantly (*p* < 0.001) increased total GSK-3β levels *versus* the siControl (Fig. 1*B*). In contrast, the inhibitory phosphorylation of GSK-3β at Ser9 (61) decreased significantly (*p* < 0.001), while the activating phosphorylation of GSK-3β at Tyr216 (62) increased significantly (*p* < 0.01) after *NDRG1* silencing (Fig. 1*B*). However, this increase in total GSK-3β levels and its activation after *NDRG1* silencing did not increase β-catenin phosphorylation at Ser33, Ser37, and Thr41. In fact, the levels of this later phosphorylation were significantly (*p* < 0.01) decreased *versus* the siControl (Fig. 1*B*). Thus, these later results additionally suggest a GSK-3β-independent mechanism regulates the phosphorylation of β-catenin at Ser33, Ser37, and Thr41. The silencing of *NDRG1* resulted in no significant (*p* > 0.05) change in total AKT levels, while significantly (*p* < 0.001) increasing its phosphorylation at Ser473 (Fig. 1*B*).

To further corroborate that *NDRG1* silencing increases GSK-3β levels and its activity, levels of the GSK-3β antagonist, FRAT1, were examined after silencing *NDRG1* and were shown to be significantly (*p* < 0.01) decreased *versus* the siControl (Fig. 1*B*). Since FRAT1 inhibits GSK-3β binding to the destruction complex, which then prevents β-catenin phosphorylation (58), a decrease of FRAT1 in *NDRG1* silenced cells should increase GSK-3β activity and increase phosphorylation of β-catenin at Ser33, Ser37, Thr41 (58). However, since a significant (*p* < 0.01) decrease in β-catenin phosphorylation at Ser33, Ser37, and Thr41 occurred after *NDRG1* silencing (Fig. 1*B*), this again indicates a GSK-3β-independent mechanism for phosphorylating these later residues on β-catenin.

Considering the possible GSK-3β-independent mechanism involved in the phosphorylation of β-catenin at Ser33, Ser37, and Thr41, *NDRG1* silencing in AsPC-1 cells demonstrated a significant (*p* < 0.001) decrease in PKCα *versus* the siControl (Fig. 1*B*). This observation was opposite to the PKCα upregulation observed after NDRG1 overexpression (Fig. 1*A*). The decrease in PKCα after *NDRG1* silencing is consistent with the significant decrease in β-catenin phosphorylation at Ser33, Ser37, and Thr41 under these conditions (Fig. 1*B*). Such alterations that lead to increased β-catenin levels could promote WNT signaling (59). Consistent with this finding, the WNT downstream effector, cyclin D1, was significantly (*p* < 0.001) increased after *NDRG1* silencing relative to VC cells (Fig. 1*B*).

These results further support the role of PKCα in the GSK-3β-independent mechanism for mediating the phosphorylation of β-catenin at Ser33, Ser37, and Thr41 induced by NDRG1 overexpression.

### *PKCα* silencing increases total β-catenin and decreases the destabilizing β-catenin phosphorylation at Ser33, Ser37, and Thr41

**Figure 2:**
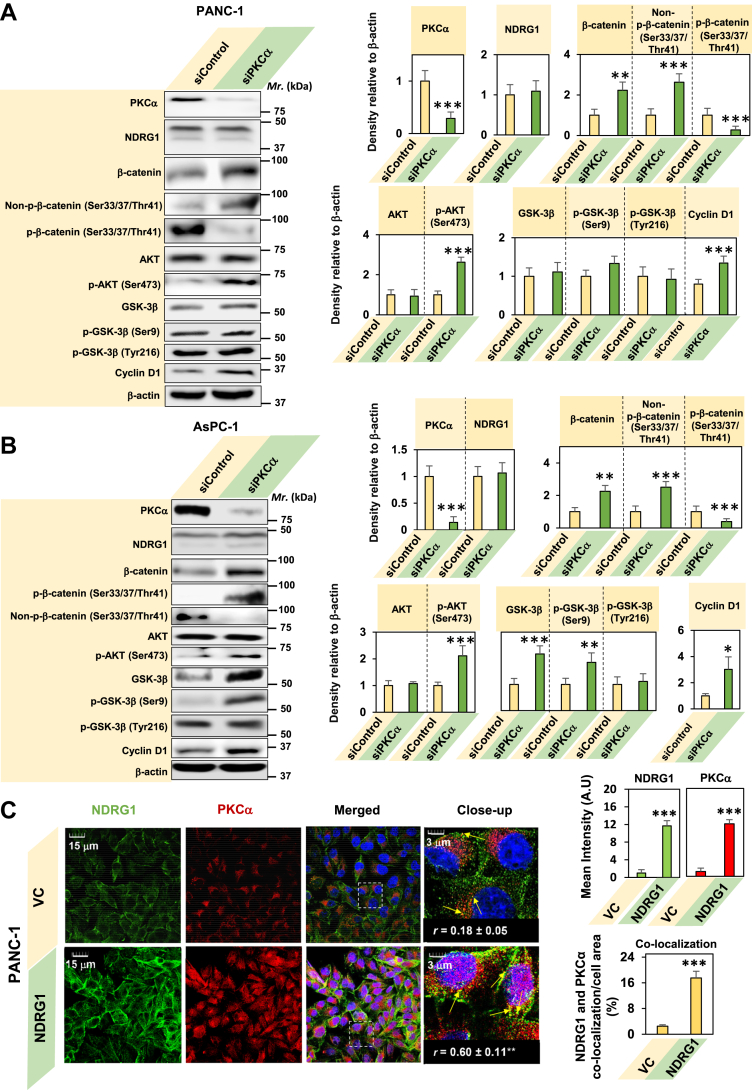
***PKCα*****silencing increases total β-catenin and decreases the destabilizing β-catenin phosphorylation at Ser33, Ser37, and Thr41,*****via*****possible association with NDRG1.***A*, *PKCα* silencing in PANC-1 cells results in an increase in β-catenin levels while having no significant effect on GSK-3β expression. PANC-1 PC cells were incubated with control medium for 24 h/37 °C and then transiently transfected with *PKCα* siRNA or non-targeting negative control siRNA (siControl) for 24 h/37 °C. Total cell protein lysate then underwent SDS–PAGE and analyzed *via* western blotting. β-actin was used as a protein-loading control. The blot is representative of three experiments, while the densitometry is mean ± SD (*n =* 3). *B*, *PKCα* silencing in AsPC-1 cells results in an increase of total β-catenin levels, while increasing the inhibitory Ser9 phosphorylation of GSK-3β. AsPC-1 PC cells were incubated with control medium for 24 h/37 °C and then transiently transfected with *PKCα* siRNA or non-targeting negative control siRNA (siControl) for 24 h/37 °C. Total cell protein lysate underwent SDS–PAGE and was analyzed *via* western blotting as in (*A*). *C*, confocal microscopy demonstrates that NDRG1 overexpression in PANC-1 cells increases the association between NDRG1 and PKCα. VC and NDRG1 overexpressing PANC-1 cells were examined for NDRG1 and PKCα expression and localization using confocal microscopy (60× objective) to demonstrate NDRG1 (*green*) and PKCα (*red*) staining. *Yellow arrows* show co-localization between NDRG1 and PKCα. The nucleus was stained by DAPI (*blue*). Images are representative of three experiments with pixel intensities and co-localization of NDRG1 and PKCα being quantitated with ImageJ using 24 cells. The Pearson correlation coefficient (*r*) was calculated using the Image J plugin, JACoP. Quantitation of the results represents the mean ± SD (*n =* 3). The scale bar = 15 μm except for the close-up image, where it is 3 μm. Statistical significance in (*A*–*C*) is denoted as ∗*p* < 0.05, ∗∗*p* < 0.01 and ∗∗∗*p* < 0.001 comparing siControl with siPKCα, or VC and NDRG1 overexpression.

To additionally investigate the functional role of PKCα in β-catenin phosphorylation, *PKCα* was silenced in PANC-1 and AsPC-1 cells using siRNA (Fig. 2, *A* and *B*). Silencing of *PKCα* resulted in a marked and significant (*p* < 0.001) decrease in PKCα levels in both PANC-1 (Fig. 2*A*) and AsPC-1 cells (Fig. 2*B*) *versus* their respective siControls. The levels of NDRG1 were not significantly (*p* > 0.05) affected by the silencing of *PKCα* in both cell-types (Fig. 2, *A* and *B*) compared to their siControls. This finding suggests that while NDRG1 overexpression acts as a positive regulator of PKCα expression (Fig. 1*A*), PKCα does not regulate NDRG1 levels.

As hypothesized above, silencing *PKCα* resulted in a significant (*p* < 0.001–0.01) increase in total and stable non-phosphorylated (Ser33, Ser37, Thr41) β-catenin levels in both PANC-1 (Fig. 2*A*) and AsPC-1 cells (Fig. 2*B*) relative to their siControls. There was also a significant (*p* < 0.001) decrease in phosphorylated β-catenin (Ser33, Ser37, Thr41) levels in both *PKCα* silenced PANC-1 (Fig. 2*A*) and AsPC-1 cells (Fig. 2*B*) *versus* the siControls. Thus, silencing *PKCα* prevents its activity in terms of phosphorylating β-catenin at Ser33, Ser37, and Thr41, resulting in increased total β-catenin that should promote WNT signaling. This suggestion was confirmed by examining expression of the key WNT downstream effector, cyclin D1, that was significantly (*p* < 0.001–0.05) increased upon silencing *PKCα versus* the siControl (Fig. 2, *A* and *B*).

As indicated previously, considerable cross-talk exists between AKT and PKCα signaling (60), with NDRG1 overexpression decreasing p-AKT (Ser473) levels and its downstream target p-β-catenin (Ser552; Fig. 1*A*). This latter effect may be mediated by the upregulation of PKCα after NDRG1 overexpression. This putative relationship was examined here by silencing *PKCα,* which significantly (*p* < 0.001) increased p-AKT (Ser473), but not total AKT levels *versus* the siControl in both PANC-1 and AsPC-1 PC cells (Fig. 2, *A* and *B*). These findings are consistent with the idea of cross-talk between PKCα and AKT, suggesting that PKCα mediates a tumor suppressive role upon NDRG1 overexpression.

Since GSK-3β classically results in the destabilizing phosphorylation (Ser33, Ser37, Thr41) of β-catenin (57), and that NDRG1 overexpression decreases GSK-3β total levels and activity (Fig. 1*A*), it was important to examine if NDRG1-induced upregulation of PKCα is the mechanism by which NDRG1 suppressed GSK-3β. Interestingly, *PKCα* silencing in PANC-1 cells had no significant (*p* > 0.05) effect on total GSK-3β levels or its inhibitory and activating phosphorylations (Ser9 and Tyr216, respectively) compared to the siControl (Fig. 2*A*). This observation suggests that GSK-3β activity in PANC-1 cells was PKCα-independent.

In contrast to the results with PANC-1 cells (Fig. 2*A*), when GSK-3β was examined in *PKCα* silenced AsPC-1 cells, the total protein levels, and inhibitory phosphorylation (Ser9) of GSK-3β were significantly (*p* < 0.001–0.01) increased *versus* the siControl (Fig. 2*B*). Previous studies examining the effect of *PKCα* knockout in mice led to similar results where total and p-Ser9 levels of GSK-3β increased relative to wild-type mice (63). The increased p-GSK-3β (Ser9) levels upon *PKCα* knockout were attributed to an increase in AKT (63), which catalyzes the inhibitory phosphorylation of GSK-3β at Ser9 (64). Therefore, the increase in p-GSK-3β (Ser9) in AsPC-1 cells may be due to the increase in AKT activation (Ser473) upon *PKCα* silencing (Fig. 2*B*). Interestingly, the activating GSK-3β phosphorylation at Tyr216 was not significantly (*p* > 0.05) altered in *PKCα* silenced AsPC-1 cells compared to the siControl (Fig. 2*B*). The different effects of *PKCα* silencing on GSK-3β observed in PANC-1 (Fig. 2*A*) and AsPC-1 cells (Fig. 2*B*) suggests the role of PKCα is cell-type specific. Nonetheless, silencing *PKCα* increased total β-catenin expression in both cell-types and also resulted in the upregulation of cyclin D1 expression *versus* the siControl (Fig. 2, *A* and *B*), suggesting its role in regulating WNT signaling.

Overall, *PKCα* silencing in Figure 2, *A* and *B* is consistent with the conclusion that PKCα downregulates β-catenin by phosphorylating it at Ser33, Ser37, and Thr41, which causes β-catenin degradation. These silencing studies also confirm the inhibitory effect of NDRG1 overexpression on the WNT/β-catenin pathway (Fig. 1*A*) is caused, at least in part, by the upregulation of PKCα.

### Confocal microscopy and co-immunoprecipitation (co-IP) demonstrate the association of NDRG1 and PKCα

The studies in Figures 1 and 2 support the hypothesis that NDRG1 inhibits the β-catenin pathway by increasing PKCα expression. As NDRG1 stabilizes and increases the expression of other proteins (*e.g.*, MIG6, androgen receptor, O^6^-methylguanine-DNA methyltransferase, *etc.*) by promoting its association with these targets (17, 65, 66), confocal microscopy studies were initiated to examine the co-localization between NDRG1 and PKCα (Fig. 2*C*). In agreement with western analysis (Fig. 1*A*), these studies demonstrated a significant (*p* < 0.001) increase in NDRG1 and PKCα intensity in NDRG1 overexpressing cells relative to VC cells (Fig. 2*C*). Furthermore, NDRG1 and PKCα had a similar distribution in cells, being predominantly perinuclear (Fig. 2*C*).

Since confocal microscopy images are captured from a single lateral focal plane, proteins present in a similar location can be co-localized (67). There was a marked and significant (*p* < 0.001) increase of co-localization between NDRG1 and PKCα in NDRG1 overexpressing cells *versus* VC cells (Fig. 2*C*), suggesting an increased association between NDRG1 and PKCα. Similarly, a significant (*p* < 0.01) increase was observed in the Pearson correlation coefficient (*r*) upon NDRG1 overexpression *versus* the VC (from 0.18 ± 0.05–0.60 ± 0.11; Fig. 2*C*).

**Figure 3:**
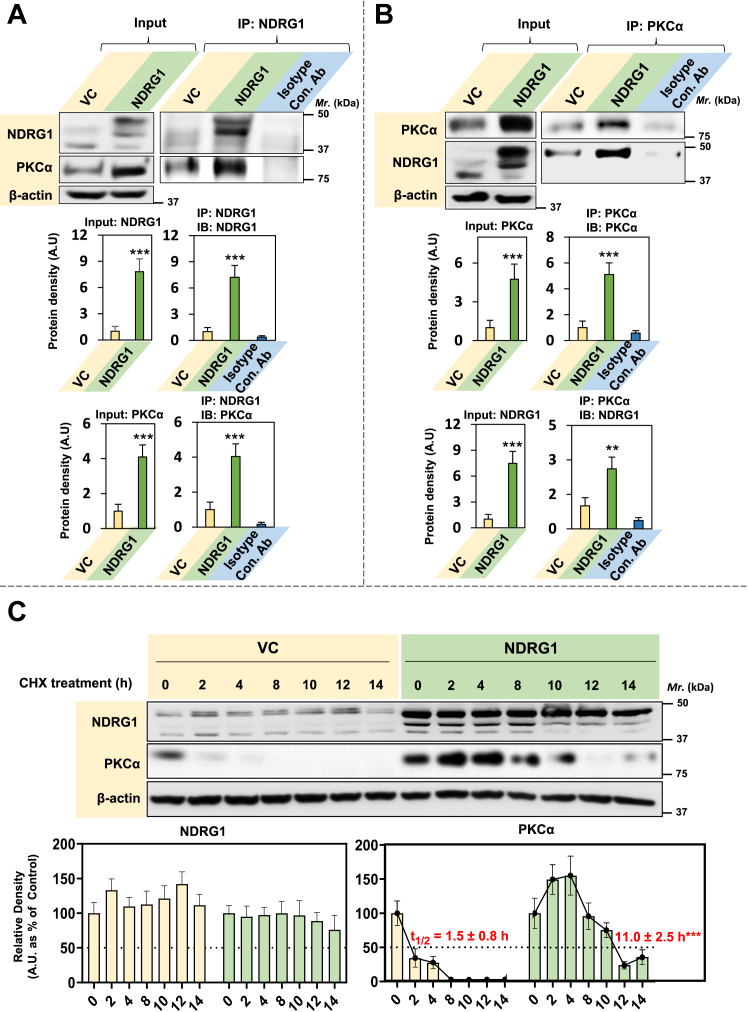
**Co-immunoprecipitation demonstrates the association of NDRG1 and PKCα, extending the half-life of PKCα.***A* and *B*, association between NDRG1 and PKCα significantly increases in NDRG1 overexpressing cells as does (*C*) PKCα half-life. PANC-1 VC and NDRG1 cells were incubated with control medium at 37 °C/24 h and then lysed using IP lysis buffer and 400 μg of whole cell lysate incubated overnight at 4 °C with either: (*A*) NDRG1 or (*B*) PKCα antibodies or an isotype control antibody (Isotype Con. Ab). Protein A/G bound to magnetic beads was then added to cell lysates and incubated for 1.5 h/4 °C. Samples were placed on a magnet to isolate antibody/protein complexes, which were then subjected to western blotting. *C*, PANC-1 VC and NDRG1 cells were preincubated with the protein synthesis inhibitor, cycloheximide (CHX; 15 μg/ml), for 1 h/37 °C and then chased for 2 to 14 h/37 °C with CHX (15 μg/ml), harvested, and western analysis performed. Densitometry is presented as the mean ± SD (*n =* 3). Statistical significance is denoted as ∗∗*p* < 0.01 and ∗∗∗*p* < 0.001 comparing NDRG1 overexpressing cells *versus* VC cells.

To further test the hypothesis that NDRG1 and PKCα associate, co-IP analysis was undertaken using PANC-1 cell model as the effects of NDRG1 are well characterized in this cell-type by our laboratory (9). The co-IP was performed using antibodies against both NDRG1 (Fig. 3*A*) or PKCα (Fig. 3*B*) to validate the interaction. An isotype control antibody (Isotype Con. Ab) was also utilized to assess the non-specific binding of immunoglobins to the protein of interest. Western analysis (input) of total cell lysates and co-IP using an NDRG1 antibody demonstrated significantly (*p* < 0.001) higher NDRG1 levels in NDRG1 overexpressing cells compared to VC cells (Fig. 3*A*). Examination of PKCα expression in NDRG1 overexpressing cells *versus* the VC also indicated significantly (*p* < 0.001) increased levels in the input and also after co-IP with an NDRG1 antibody (Fig. 3*A*).

Western analysis of the input and co-IP using a PKCα antibody demonstrated significantly (*p* < 0.001) higher PKCα levels in NDRG1 overexpressing cells (Fig. 3*B*). Furthermore, there was a significant (*p* < 0.001–0.01) increase of NDRG1 in the input and after co-IP with the PKCα antibody (Fig. 3*B*). For the co-IP investigations in Figure 3, *A* and *B*, the Isotype Con. Ab demonstrated very low non-specific binding and indicated the high specificity of the antibody used for IP of the target protein.

In summary, these results in Figure 3, *A* and *B* indicate a marked increase in the association of NDRG1 and PKCα in NDRG1 overexpressing cells *versus* the VC and support the evidence of this interaction from confocal microscopy in Figure 2*C*.

### PKCα half-life is increased upon NDRG1 overexpression

It can be hypothesized from the studies in Figures 2*C* and 3, *A* and *B* that NDRG1 associates with PKCα, stabilizes PKCα, and potentially increases its half-life, as reported for the association of NDRG1 with other proteins (17, 65, 66). To examine this, the half-life of PKCα in PANC-1 NDRG1-overexpressing cells was compared to that in VC cells using the protein synthesis inhibitor, cycloheximide (CHX; Fig. 3*C*). Western blot analysis clearly demonstrated the higher NDRG1 levels in the NDRG1 overexpressing clone *versus* the VC. However, upon incubation with CHX (15 μg/ml), there was no significant (*p* > 0.05) change in NDRG1 levels for incubations up to 14 h in both cell-types relative to the 0 h control (Fig. 3*C*). In marked contrast, examining VC cells a pronounced decrease in PKCα expression was observed after an incubation of 2 h *versus* the 0 h control. On the other hand, examining NDRG1 overexpressing cells, a decrease in PKCα levels relative to the 0 h control was only apparent after a 10 h incubation (Fig. 3*C*). Calculation of the half-life of PKCα from these data demonstrated that it was significantly (*p* < 0.001) increased from 1.5 ± 0.8 h (3) in VC cells to 11.0 ± 2.5 h (3) in NDRG1-overexpressing cells, respectively (Fig. 3*C*). In conclusion, the protein half-life of PKCα is markedly increased upon NDRG1 expression in PANC-1 cells, indicating PKCα stabilization.

### NDRG1 overexpression results in a significant association between PKCα and β-catenin and a decrease in β-catenin expression

**Figure 4:**
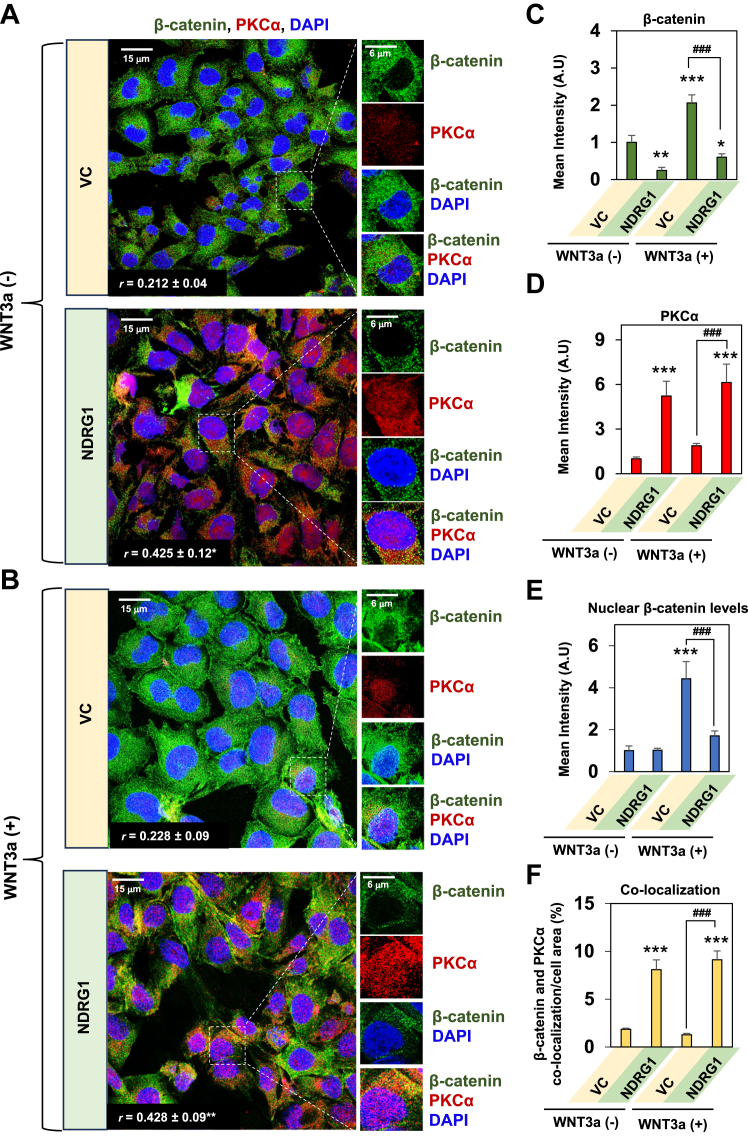
**NDRG1 overexpression results in a significant association between PKCα and β-catenin and a decrease in β-catenin expression in the presence or absence of WNT3a.***A*–*F*, NDRG1 overexpression in PANC-1 cells significantly increases co-localization between β-catenin and PKCα in the presence and absence of WNT3a. *A* and *B*, VC and NDRG1 overexpressing PANC-1 cells were incubated for 24 h/37 °C in the presence and absence of WNT3a (100 ng/ml). The cells were then examined for β-catenin (*green*) and PKCα (*red*) expression and co-localization (*yellow*) using confocal immunofluorescence microscopy. Cell nuclei were stained by DAPI (*blue*). Images are representative of three experiments and the quantitative analysis of intensities presented for: (*C*) β-catenin; (*D*) PKCα; (*E*) nuclear β-catenin; and (*F*) co-localization between PKCα and β-catenin. Studies were performed using a 60× objective at the same acquisition setting with Olympus Fluoview FV3000 software. ImageJ software was used for the analysis and the Pearson correlation coefficient (*r*) was calculated using the Image J plugin, JACoP. Quantitative analyses are presented as the mean ± SD (*n =* 3). Analysis of pixel intensity and co-localization were performed using 24 cells. The scale bar is 15 μm except for the close-up images, where it is 6 μm. Statistical significance is denoted as ∗*p* < 0.05, ∗∗*p* < 0.01 and ∗∗∗*p* < 0.001 comparing NDRG1 overexpressing PANC-1 cells in the presence or absence of WNT3a to VC cells in the absence of WNT3a; or ^###^*p* < 0.001 comparing NDRG1 overexpressing PANC-1 cells to VC cells in the presence of WNT3a.

Apart from the association of NDRG1 with PKCα demonstrated previously (Figs. 2*C* and 3, *A* and *B*), PKCα has been reported to directly bind to the armadillo repeat sequences of β-catenin (27), Together, these observations suggest a complex interactome of proteins that required further investigation that was initially performed using confocal microscopy (Fig. 4, *A*–*F*).

Studies were first designed to investigate the association between PKCα with β-catenin using NDRG1 overexpressing PANC-1 cells *versus* their VC counterparts. In these experiments, cells were incubated for 24 h in the absence (Fig. 4*A*) or presence (Fig. 4*B*) of the WNT ligand, WNT3a (100 ng/ml), which stimulates nuclear translocation of β-catenin (68). In the absence of WNT3a in VC cells, β-catenin demonstrated predominantly cytoplasmic expression, while little PKCα was apparent (Fig. 4*A*). Examining PANC-1 cells with NDRG1 overexpression in the absence of WNT3a, there was a significant (*p* < 0.01) decrease in total cellular β-catenin levels (Fig. 4*C*) and a pronounced and significant (*p* < 0.001) increase in PKCα expression (Fig. 4*D*). These latter results confirmed the findings of western analysis under analogous conditions (Fig. 1*A*).

In the absence of WNT3a, no significant (*p* > 0.05) change in nuclear β-catenin was observed after NDRG1 overexpression *versus* the VC (Fig. 4*E*). Additionally, without WNT3a, a significant (*p* < 0.001) increase in the co-localization of β-catenin with PKCα was demonstrated after NDRG1 overexpression *versus* VC cells (Fig. 4*F*). The co-localization of PKCα (red) with DAPI (blue) resulting in purple nuclei (Fig. 4*A*) indicated this protein demonstrated some nuclear translocation after NDRG1 overexpression. Previous studies under several conditions have also demonstrated PKCα translocation to the nucleus (69, 70).

As expected from its role in activating WNT signaling (68), upon incubating VC cells with WNT3a, there was a significant (*p* < 0.001) increase in total (Fig. 4, *B*, *C*, and *E*) and nuclear β-catenin expression (Fig. 4*E*) *versus* VC cells without WNT3a. The addition of WNT3a also resulted in a slight increase (*p* > 0.05) in PKCα expression in VC cells relative to VC cells incubated without WNT3a (Fig. 4*D*). Some limited nuclear co-localization of PKCα, β-catenin, and DAPI was evident (white fluorescence) in VC cells treated with WNT3a relative to VC cells without WNT3a (Fig. 4*B*). Only very sparse co-localization (yellow) of β-catenin and PKCα was demonstrated in VC cells after incubation with WNT3a, but this was not significantly (*p* > 0.05) different to VC cells without WNT3a (Fig. 4, *B* and *F*).

After the addition of WNT3a to NDRG1 overexpressing cells there was a significant (*p* < 0.001) decrease in total (Fig. 4, *B* and *C*) and nuclear β-catenin levels (Fig. 4, *B* and *E*) *versus* the VC incubated with WNT3a. In the presence WNT3a, there was also a significant (*p* < 0.001) increase in PKCα in NDRG1 overexpressing cells relative to VC cells (Fig. 4, *A* and *D*). Due to the decrease in nuclear β-catenin levels after NDRG1 overexpression in the presence of WNT3a, no marked co-localization (white) was observed between DAPI (blue), PKCα (red), and β-catenin (green; Fig. 4, *B* and *E*). However, in the presence of WNT3a and after NDRG1 overexpression, there was a pronounced and significant (*p* < 0.001) increase in co-localization (yellow) between PKCα and β-catenin relative to VC cells (Fig. 4, *B* and *F*).

In summary, confocal microscopy in Figure 4 demonstrated that NDRG1 overexpression increased PKCα expression in the presence and absence of WNT3a and resulted in co-localization between PKCα and β-catenin. Further, NDRG1 overexpression demonstrated anti-oncogenic activity resulting in decreased total and nuclear β-catenin levels relative to VC cells in the presence of WNT3a.

### NDRG1 co-localizes with β-catenin upon NDRG1 overexpression and decreases total β-catenin levels and its nuclear localization in the presence of WNT3a

**Figure 5:**
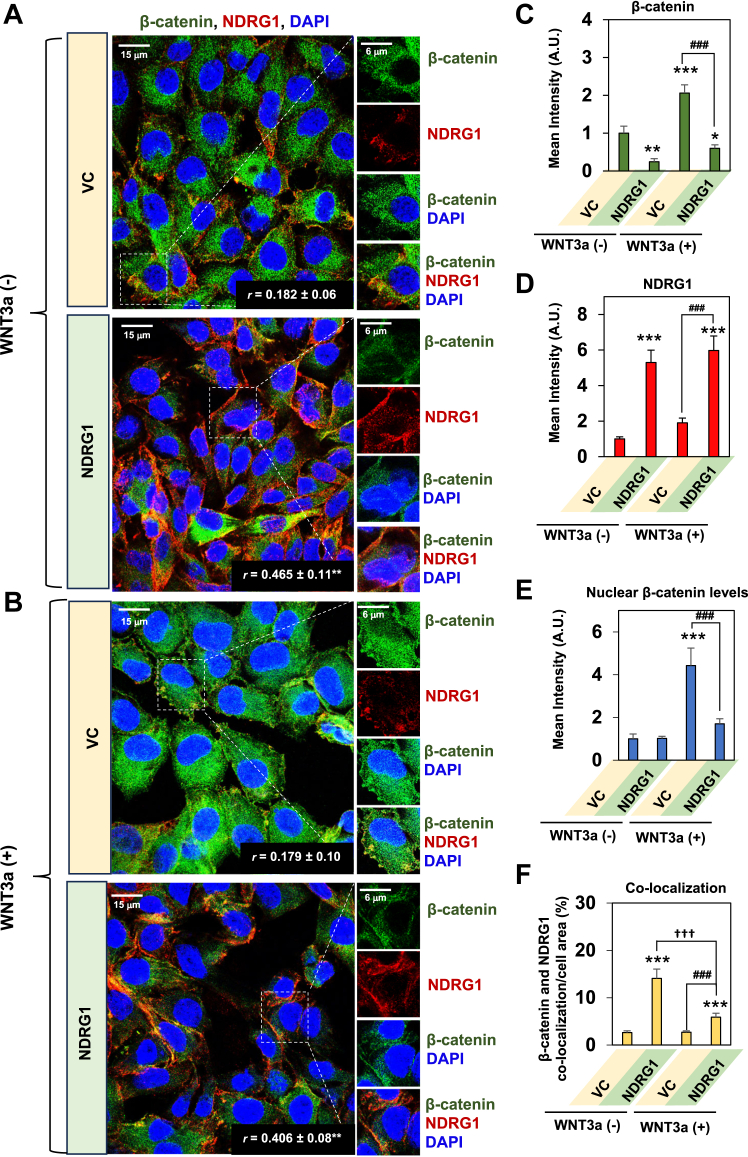
**NDRG1 co-localizes with β-catenin upon NDRG1 overexpression and decreases total β-catenin levels and its nuclear localization in the presence or absence of WNT3a.***A*–*F*, NDRG1 overexpression in PANC-1 cells significantly increases the co-localization between β-catenin and NDRG1 in the presence of WNT3a, but particularly its absence. *A* and *B*, VC and NDRG1 overexpressing PANC-1 cells were incubated for 24 h/37 °C in the presence and absence of WNT3a (100 ng/ml). The cells were then examined for β-catenin (*green*) and NDRG1 (*red*) expression and co-localization (*yellow*) using confocal immunofluorescence microscopy. Cell nuclei were stained by DAPI (*blue*). Studies were performed using a 60× objective at the same acquisition setting with Olympus Fluoview FV3000 software. Images are representative of three experiments and the quantitative analysis of intensities presented for: (*C*) β-catenin (reuse from Fig. 4*C*); (*D*) NDRG1; (*E*) nuclear β-catenin (reuse from Fig. 4*E*); and (*F*) co-localization between β-catenin and NDRG1. ImageJ software was used for the analysis and the Pearson correlation coefficient (*r*) was calculated using the Image J plugin, JACoP. Quantitative analyses are presented as the mean ± SD (*n =* 3). Analysis of pixel intensity and co-localization were performed using 24 cells. The scale bar is 15 μm except for the inset images, where it is 6 μm. Statistical significance is denoted as ∗*p* < 0.05, ∗∗*p* < 0.01 and ∗∗∗*p* < 0.001 comparing NDRG1 overexpressing PANC-1 cells or VC cells in the presence or absence of WNT3a to VC cells in the absence of WNT3a; ^###^*p* < 0.001 comparing NDRG1 overexpressing PANC-1 cells to VC cells in the presence of WNT3; or ^†††^*p* < 0.001 comparing NDRG1 overexpressing PANC-1 cells in the absence of WNT3a to NDRG1 overexpressing PANC-1 cells in the presence of WNT3a.

In addition to the interaction between PKCα and β-catenin (Fig. 4), NDRG1 has also been described as associated with β-catenin in esophageal squamous carcinoma cells (71). To examine if this also occurs in PC cells, the cellular distribution and association between β-catenin (green) and NDRG1 (red) was examined by confocal microscopy in the absence and presence of WNT3a in PANC-1 VC and NDRG1 overexpressing cells (Fig. 5, *A*–*F*). Investigating VC cells in the absence of WNT3a, there were appreciable total β-catenin levels (Fig. 5, *A* and *C*) and low NDRG1 expression (Fig. 5, *A* and *D*) that led to very limited cytoplasmic co-localization (yellow; Fig. 5, *A* and *F*). Overexpression of NDRG1 in the absence of WNT3a resulted in significantly (*p* < 0.01) decreased total cellular β-catenin expression *versus* the VC (Fig. 5, *A* and *C*). In the absence of WNT3a, NDRG1 overexpression led to a significant (*p* < 0.001) increase in cytoplasmic and membrane co-localization of NDRG1 and β-catenin relative to VC cells (Fig. 5, *A* and *F*).

The addition of WNT3a to VC cells, significantly (*p* < 0.001) increased total (Fig. 5, *B* and *C*) and nuclear β-catenin (Fig. 5, *B* and *E*) expression relative to VC cells in the absence of WNT3a, as observed in Figure 4, *B*, *C*, and *E*. However, examining VC cells in the presence of WNT3a, there was no significant (*p* > 0.05) change in the co-localization of NDRG1 and β-catenin relative to the VC cells in the absence of WNT3a (Fig. 5, *B* and *F*). The overexpression of NDRG1 in cells incubated with WNT3a resulted in significantly (*p* < 0.001) decreased total β-catenin (Fig. 5, *B* and *C*) and nuclear β-catenin levels (Fig. 5, *B* and *E*) *versus* the VC in the presence of WNT3a.

Notably, comparing NDRG1 overexpressing and VC cells in the presence of WNT3a, despite the significant (*p* < 0.001) decrease in total cellular β-catenin levels (Fig. 5, *B* and *C*), the co-localization of NDRG1 and β-catenin was significantly (*p* < 0.001) enhanced and was predominantly at the plasma membrane (Fig. 5*B*). This observation can be explained by our previous studies demonstrating the ability of NDRG1 to stabilize β-catenin at the plasma membrane as part of the adherens junction, which maintains cellular adhesion and inhibits the EMT (7). However, the co-localization between β-catenin and NDRG1 was significantly (*p* < 0.001) less pronounced in the presence of WNT3a than in its absence (Fig. 5*F*).

**Figure 6:**
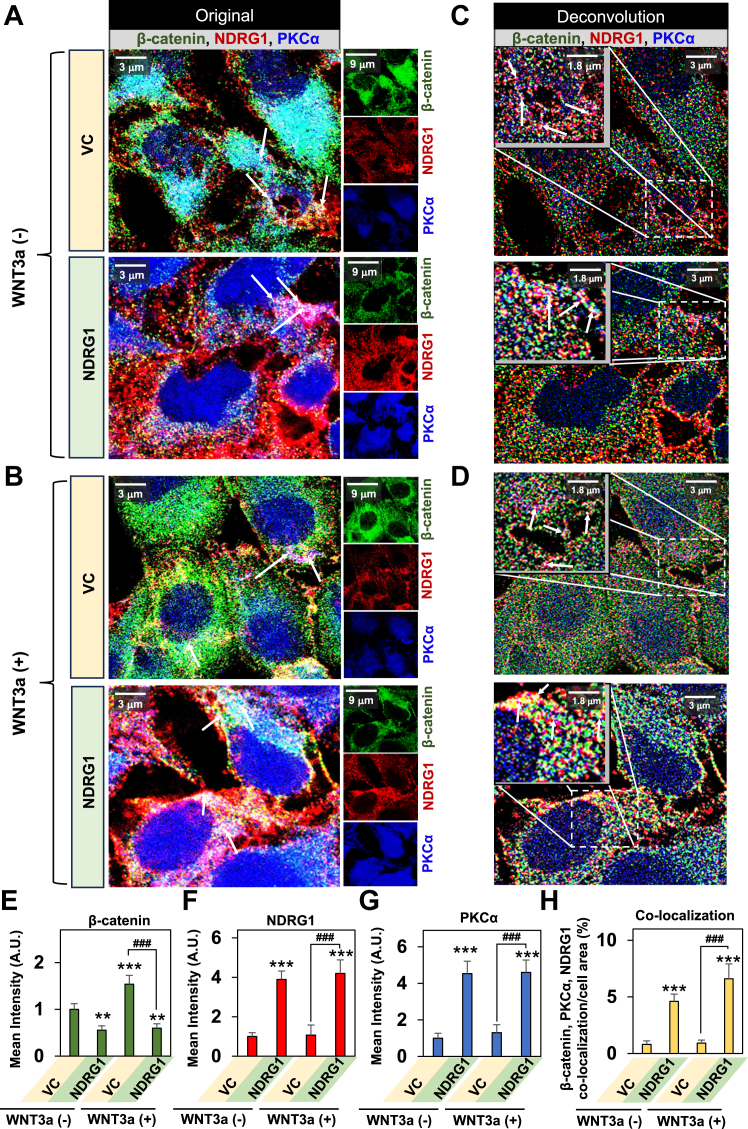
**Triple co-localization of β-catenin, NDRG1, and PKCα demonstrates the formation of a possible metabolon, decreasing β-catenin levels and nuclear localization.***A*–*H*, NDRG1 overexpression in PANC-1 cells significantly increases the co-localization between β-catenin, NDRG1, and PKCα. *A* and *B*, VC and NDRG1 overexpressing PANC-1 cells were incubated for 24 h/37 °C in the presence and absence of WNT3a (100 ng/ml). The cells were then examined for β-catenin (*green*), NDRG1 (*red*), and PKCα (*blue*) expression and triple co-localization (*white*) using confocal immunofluorescence microscopy. Studies were performed using a 100× objective at the same acquisition setting with Olympus Fluoview FV3000 software. Images were digitally magnified for better demonstration of the possible co-localization. The scale bar = 3 μm for all images except for the inset images, where the scale bar = 9 μm. *C* and *D*, deconvolution analysis was then implemented on the images in (*A* and *B*) using Olympus CellSens imaging software. White arrows demonstrate triple co-localization and possible formation of the metabolon. The scale bar = 3 μm for all deconvolution images except for the inset images, where the scale bar = 1.8 μm. In all studies, the images are representative of three experiments, and the quantitative analysis of the pixel intensities is shown for (*E*) β-catenin; (*F*) NDRG1; (*G*) PKCα; and (*H*) Co-localization between β-catenin, NDRG1, and PKCα. Quantitative analyses were performed using ImageJ software and were presented as the mean ± SD (*n =* 3). Analysis of pixel intensity and co-localization were performed using 24 cells. Statistical significance is denoted as ∗∗*p* < 0.01 and ∗∗∗*p* < 0.001 comparing NDRG1 overexpressing PANC-1 cells in the presence and absence of WNT3a to VC cells in the absence of WNT3a; or ###*p* < 0.001 comparing NDRG1 overexpressing PANC-1 cells to VC cells in the presence of WNT3a.

Considering the association between PKCα and NDRG1 (Figs. 2*C* and 3, *A* and B), PKCα and β-catenin (Fig. 4, *A*, *B*, and *F*), and NDRG1 and β-catenin (Fig. 5, *A*, *B*, and *F*), it was considered if all three proteins may associate (Fig. 6, *A*–*H*). In fact, the triple co-localization (white) of PKCα (blue), NDRG1 (red), and β-catenin (green) was significantly (*p* < 0.001) increased after NDRG1 overexpression, in the absence and especially the presence of WNT3a (Fig. 6*H*). These studies suggest an association between these three proteins that occurred primarily in the perinuclear region of the cell.

Overall, confocal microscopy in Figure 4, Figure 5, Figure 6 demonstrates that NDRG1 overexpression decreased total β-catenin, with co-localization of β-catenin with NDRG1 being increased in the presence of WNT3a and particularly its absence. The addition of WNT3a to NDRG1-expressing cells enabled the identification of β-catenin and NDRG1 co-localization at the plasma membrane. Finally, an association between PKCα, NDRG1, and β-catenin was identified suggesting a potential metabolon.

### Association of PKCα and β-catenin, but also NDRG1 and β-catenin upon NDRG1 overexpression

**Figure 7:**
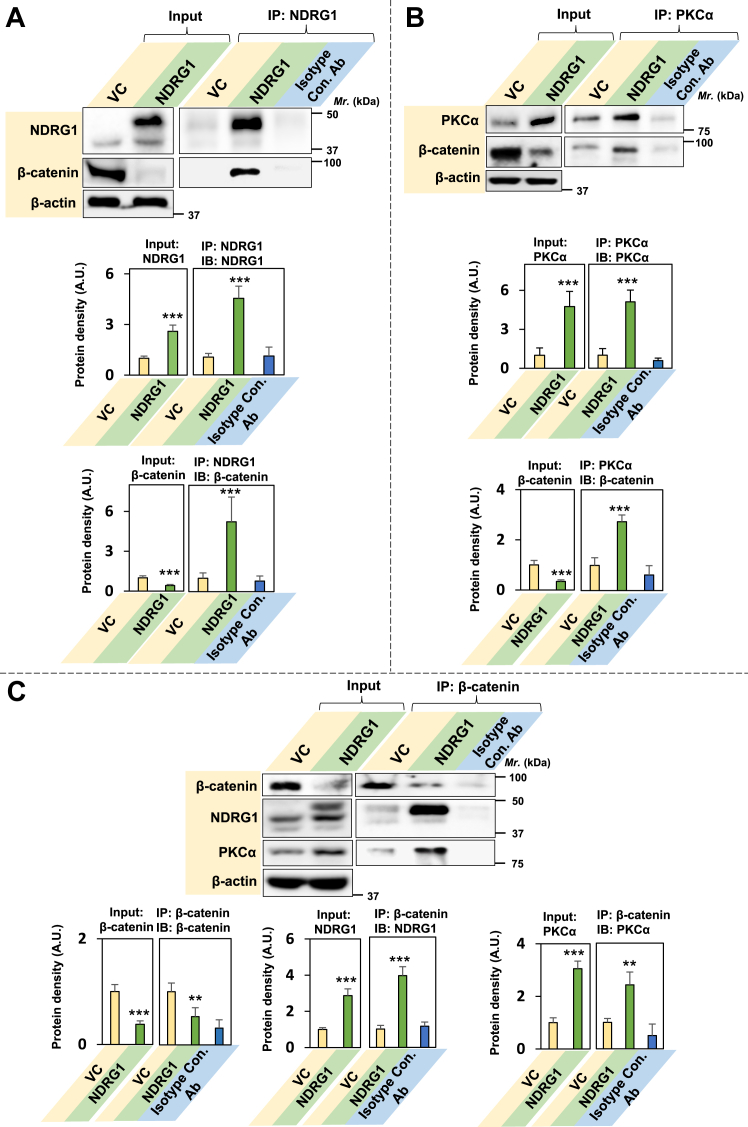
**Association of PKCα and β-catenin, but also NDRG1 and β-catenin upon NDRG1 overexpression.***A*–*C*, NDRG1 and β-catenin and also NDRG1 and PKCα associate upon NDRG1 overexpression. PANC-1 VC and NDRG1 cells were incubated with a control medium at 37 °C for 24 h and then lysed using IP lysis buffer and 400 μg of whole cell lysate incubated overnight at 4 °C with either: (*A*) NDRG1, (*B*) PKCα, or (*C*) β-catenin antibodies or a rabbit isotype control antibody (Isotope Con. Ab). Protein A/G bound to magnetic beads was then added to cell lysates and incubated for 1.5 h/4 °C. Samples were placed on a magnet to isolate antibody/protein complexes, which were then subjected to western blotting. Densitometry is presented as the mean ± SD (*n =* 3). Statistical significance is denoted as ∗∗∗*p* < 0.001 comparing PANC-1 NDRG1 *versus* VC cells.

Building upon the findings in Figure 4, Figure 5, Figure 6, to further examine the interaction between NDRG1 with β-catenin and also PKCα and β-catenin, co-IP was performed using PANC-1 VC and N1 cells (Fig. 7, *A*–*C*). As shown previously, the input demonstrated that after NDRG1 overexpression there was significant (*p* < 0.001) upregulation of NDRG1 and PKCα levels, as well as (*p* < 0.001) downregulation of β-catenin relative to VC cells (Fig. 7, *A*–*C*). Co-IP using an NDRG1 antibody demonstrated a marked and significant (*p* < 0.001) increase in β-catenin in the immunoprecipitate in PANC-1 N1 cells relative to VC cells (Fig. 7*A*).

Studies with co-IP using a PKCα antibody demonstrated a pronounced and significant (*p* < 0.001) increase in β-catenin levels in the immunoprecipitate from PANC-1 N1 cells *versus* VC cells (Fig. 7*B*). Co-IP implementing a β-catenin antibody demonstrated a significant (*p* < 0.001–0.01) increase of NDRG1 and PKCα in the immunoprecipitate from PANC-1 N1 cells *versus* VC cells (Fig. 7*C*). For all co-IP investigations in Figure 7, *A*–*C*, the Isotype Con. Ab demonstrated very low non-specific binding and indicated the high specificity of the antibody used for IP of the target protein.

Collectively, these data in Figure 7 together with the confocal microscopy results in Figure 4, Figure 5, Figure 6 are evidence of an association between NDRG1 and β-catenin and also PKCα and β-catenin. The 3 latter proteins potentially form a metabolon that could be important for facilitating their catalytic activity.

### Pharmacological induction of NDRG1 mimics genetic overexpression of this protein to upregulate PKCα and decrease β-catenin levels

Metastasis is the major killer in cancer (72, 73) and NDRG1 is a potent metastasis suppressor in multiple cancers (1, 2, 3, 4, 5, 6, 7, 8, 9, 10). Understanding the anti-oncogenic mechanism of action of NDRG1 is important regarding the development of innovative drugs in our laboratory that induce its expression (4, 44). Anti-cancer agents of the di-2-pyridylketone thiosemicarbazone class *e.g.*, di-2-pyridylketone-4,4-dimethyl-3-thiosemicarbazone (Dp44mT), clinically trialed di-2-pyridylketone-4-cyclohexyl-4-methyl-3-thiosemicarbazone (DpC), and (*E*)-3-phenyl-1-(2-pyridinyl)-2-propen-1-one 4,4-dimethyl-3-thiosemicarbazone (PPP44mT) have demonstrated activity to upregulate NDRG1 in cancer cells (9, 14, 15, 16, 17, 18, 34, 35, 36, 37, 38, 39, 40, 41, 42) with this class of agents being under development as anti-cancer drugs (74). These pharmacological NDRG1-inducing drugs are small molecular weight ligands that achieve this activity through their ability to bind tumor cell iron (33, 37, 38, 39, 40, 41, 74), leading to the activation of the transcription factor, hypoxia-inducible factor-1α, which transactivates *NDRG1* (17, 75).

**Figure 8:**
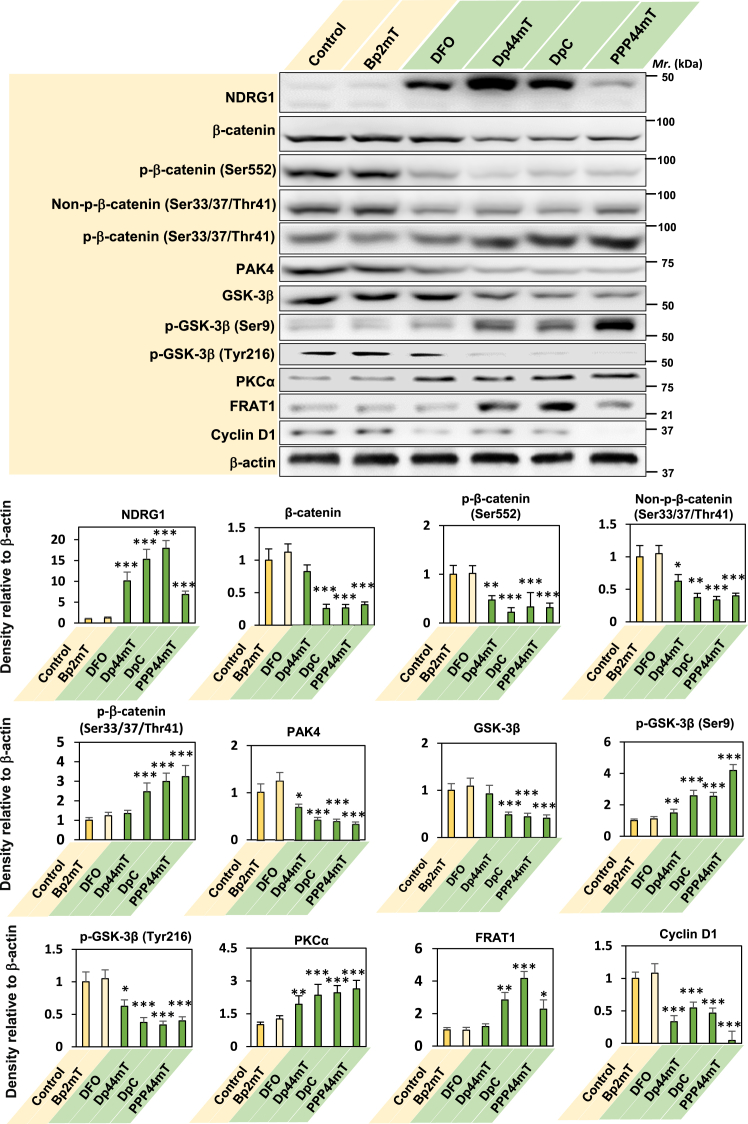
**Pharmacological induction of NDRG1 mimics genetic overexpression of this protein leading to the upregulation of PKCα and downregulation of β-catenin expression.** PANC-1 PC cells were incubated for 24 h/37 °C with control medium, Bp2mT (5 μM), DFO (100 μM) Dp44mT (5 μM), DpC (5 μM), or PPP44mT (5 μM). Total cell protein lysate then underwent SDS–PAGE and was analyzed *via* western blotting. β-actin was used as a protein-loading control. The blot is representative of three experiments, while the densitometry is mean ± SD (*n =* 3). Statistical significance is denoted as ∗*p* < 0.05, ∗∗*p* < 0.01 and ∗∗∗*p* < 0.001 relative to the Control.

Considering the ability of NDRG1 overexpression to upregulate PKCα expression and inhibit WNT signaling (Fig. 1*A*), it was important to understand if analogous activity could be identified after treatment of PC cells with the NDRG1-inducing thiosemicarbazones (Fig. 8). In these studies, PANC-1 cells were incubated for 24 h/37 °C with either a control medium or this medium containing the NDRG1-inducing thiosemicarbazones, namely Dp44mT (5 μM), DpC (5 μM), or PPP44mT (5 μM). A number of controls were also utilized, including the negative control compound for the thiosemicarbazones, namely 2-benzoylpyridine-2-methylthiosemicarbazone (Bp2mT; 5 μM). This agent has been specifically designed by our laboratory not to bind iron or other metals (76), and thus, cannot upregulate NDRG1 and does not possess biological activity (17, 18, 19, 33, 35, 41, 74). A positive control was also included, namely desferrioxamine (DFO; 100 μM), which is a well-known iron-binding ligand that is clinically used for the treatment of iron overload disease (77, 78) that upregulates NDRG1 (33, 74). The concentration of DFO used was higher herein due to its membrane permeability being limited (79) and considerably lower than the thiosemicarbazones (37).

Examining protein expression using western analysis, all agents except the negative control, Bp2mT, induced a significant (*p* < 0.001) increase in NDRG1 expression *versus* the control (Fig. 8). As observed using genetic NDRG1 overexpression (Fig. 1*A*), all NDRG1-inducing thiosemicarbazones significantly (*p* < 0.001–0.01) decreased total β-catenin, β-catenin phosphorylation at Ser552 (that is important for transcriptional activation (46)), and non-phosphorylated β-catenin at Ser33, Ser37, and Thr41, *versus* the control (Fig. 8). The phosphorylation of β-catenin at Ser33, Ser37, and Thr41 was significantly (*p* < 0.001) increased relative to the control by Dp44mT, DpC and PPP44mT. Generally, the positive control, DFO, was less effective than the thiosemicarbazones at regulating β-catenin and its phosphorylation, and this may relate to its poor cellular permeability (37, 79). Furthermore, PAK4, which is involved in the stability and nucleo-cytoplasmic translocation of β-catenin (53, 54), was significantly (*p* < 0.001–0.05) downregulated by DFO, Dp44mT, DpC, and PPP44mT, as demonstrated for genetic NDRG1 overexpression (Fig. 1*A*). Overall, the pharmacological induction of NDRG1 by the thiosemicarbazones resulted in similar effects on the regulation of β-catenin as genetic NDRG1 overexpression.

Total GSK-3β protein levels were significantly (*p* < 0.001) decreased by Dp44mT, DpC, and PPP44mT *versus* the control, while its inhibitory (Ser9) and activating (Tyr216) phosphorylation were significantly (*p* < 0.001–0.05) increased and decreased, respectively, by DFO, Dp44mT, DpC, and PPP44mT *versus* the control (Fig. 8). These later results suggest GSK-3β inactivation after incubation with the NDRG1-inducing agents, with this finding being analogous to those observed for genetic NDRG1 overexpression (Fig. 1*A*). Furthermore, these findings suggest GSK-3β was not responsible for the significant (*p* < 0.001) increase in phosphorylation of β-catenin at Ser33, Ser37, and Thr41 by Dp44mT, DpC, and PPP44mT (Fig. 8). As described for genetically overexpressed NDRG1, these observations are consistent with a GSK-3β-independent mechanism of β-catenin phosphorylation at Ser33, Ser37, and Thr41 potentially mediated by the significant (*p <* 0.001–0.01) upregulation of PKCα expression by DFO, Dp44mT, DpC, and PPP44mT relative to the control. As also observed with genetic upregulation of NDRG1 (Fig. 1*A*), expression of FRAT1 that prevents the association of GSK-3β with the destruction complex (58), was also significantly (*p <* 0.001–0.05) upregulated by Dp44mT, DpC, and PPP44mT *versus* the control (Fig. 8).

In agreement with the upregulation of NDRG1 and PKCα and downregulation of β-catenin expression by Dp44mT, DpC, and PPP44mT that could suppress WNT signaling, the downstream effector of this pathway, cyclin D1, was significantly (*p* < 0.001) downregulated by these agents *versus* the control (Fig. 8). Overall, the NDRG1-inducing agents mimicked genetic NDRG1 overexpression resulting in the upregulation of PKCα and multiple alterations in the expression of WNT pathway proteins that inhibit downstream WNT signaling.

## Discussion

PC is a highly belligerent and aggressive disease (28, 29, 80), where existing treatments are poorly effective. As such, understanding pro-oncogenic molecular pathways, for example, WNT signaling that plays a role in the pathogenesis of PC (30) and the identification of new anti-oncogenic targets, is an important research aim. Aberrant WNT/β-catenin signaling is well known in PC (22), with the metastasis suppressor, NDRG1, antagonizing this and other oncogenic signaling pathways in multiple cancer cell-types (7, 13), including PC cells and tumors (35, 47). However, the mechanisms involved remain incompletely characterized and were the subject of this investigation.

Phosphorylation of β-catenin at Ser33, Ser37, and Thr4 is a major control point in the WNT pathway that is classically mediated by GSK-3β, which results in proteasome degradation of β-catenin (57). The current work demonstrated that the critical phosphorylation of β-catenin at Ser33, Ser37, and Thr41 in PC cells was independent of GSK-3β after NDRG1 overexpression. Considering a previous report that PKCα could phosphorylate β-catenin at these sites (27), PKCα was then hypothesized to be the kinase responsible, with this conclusion being supported by NDRG1 overexpression and silencing studies in two PC cell-types. These results suggested that after NDRG1 overexpression, PKCα phosphorylated β-catenin at Ser33, Ser37, and Thr41 leading to downregulation of β-catenin. Considering the mechanism involved, NDRG1 was shown to associate with PKCα and stabilize it to facilitate its catalytic function.

Previous investigations have reported a direct interaction of NDRG1 with β-catenin (71), with PKCα interacting with the armadillo repeat sequences of β-catenin (27). Our studies have demonstrated that PKCα associates with β-catenin, and additionally for the first time, NDRG1. An interaction of NDRG1 and β-catenin was also evident, suggesting the formation of a metabolon, with this study being the first to indicate all three proteins (NDRG1, PKCα, and β-catenin) associated. As observed for many other molecules (17, 81, 82, 83, 84, 85), the formation of a metabolon can promote the activity of these proteins and ensure effective catalytic processing. Generally, it is known that proteins can associate and interact with one another on a genome-wide scale (86) to maintain stability (87), and can facilitate biological signaling even after the failure of some of their components (88).

**Figure 9:**
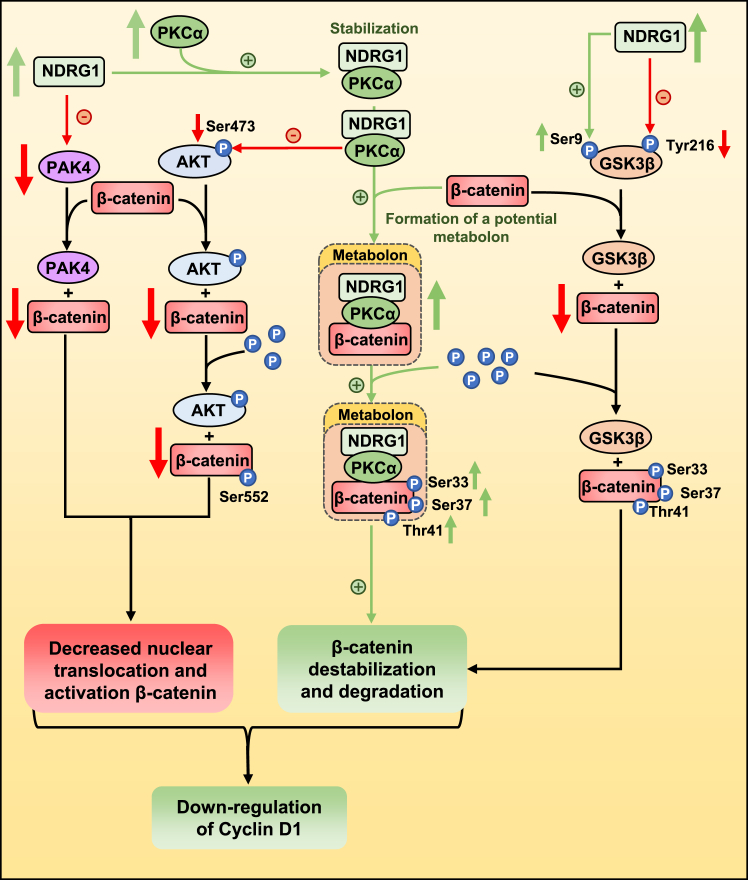
**Schematic demonstrating that overexpression of the metastasis suppressor, NDRG1, inhibits WNT/β-catenin signaling by multi-modal mechanisms involving metabolon formation in PC cells.** As an important part of this mechanism, the formation of a metabolon occurs with an association occurring between NDRG1, PKCα, and β-catenin. Expression of NDRG1 and its association with PKCα leads to the stabilization of PKCα that promotes the interaction of PKCα with β-catenin. Due to the latter association, PKCα increases the phosphorylation of β-catenin at Ser33, Ser37, and Thr41, which leads to β-catenin destabilization and degradation. NDRG1 overexpression also decreases GSK-3β protein levels and results in an increase in the inhibitory phosphorylation of GSK-3β (Ser9) and a decrease in the activation phosphorylation of GSK-3β at Tyr216. Levels of the nucleo-cytoplasmic shuttling protein, PAK4, are also decreased by NDRG1 expression, which prevents β-catenin transport to the nucleus. NDRG1 overexpression increases PKCα expression that then reduces AKT activation by decreasing its phosphorylation at Ser473. This latter effect results in the observed decrease in the AKT-mediated phosphorylation of β-catenin at Ser552 that decreasing its transcriptional activity. Together, these multi-modal effector mechanisms of NDRG1 inhibit WNT signaling in PC cells and the expression of the key downstream effector, cyclin D1.

As we show herein, NDRG1 associates with PKCα upon NDRG1 expression resulting in PKCα stabilization (*i.e.*, increased PKCα half-life). We have also demonstrated that NDRG1 overexpression increases phosphorylation of PKCα/β II (Thr638/641; Fig. S1*A*), which is important for maintaining PKCα catalytic competence (89). However, there was no change in the ratio of p-PKCα/β to total PKCα (Fig. S1*A*), indicating the increase in p-PKCα/β is due to the elevation in PKCα protein levels. Silencing of *NDRG1* had the opposite effect to NDRG1 overexpression, decreasing PKCα phosphorylation and total levels of PKCα, while having no effect on their ratio (Fig. S1*B*). Nonetheless, the current study demonstrates the upregulation of PKCα promotes association with β-catenin, leading to its phosphorylation at Ser33, Ser37, and Thr41, resulting in the downregulation of β-catenin that is anti-oncogenic (Fig. 9).

Our previous investigations examining the effect of NDRG1 overexpression on WNT signaling in prostate and colon cancer cells demonstrated a distinctly different mechanism (13) to that reported herein. In fact, NDRG1 expression increased total and non-phosphorylated (Ser33, Ser37, and Thr41) β-catenin, while there was a decrease in the phosphorylation of β-catenin at Ser33, Ser37, and Thr41 (13). The mechanism of inhibiting β-catenin phosphorylation in these latter cell-types was not mediated by significant alterations in GSK-3β total levels nor its activation but involved NDRG1-mediated upregulation of FRAT1, and downregulation of PAK4 (13). In this prior work, the effects in prostate and colon cancer cells were consistent with the role of FRAT1 in preventing the association of GSK-3β with the destruction complex, while the downregulation of PAK4 inhibited nuclear translocation of β-catenin (13). These effects of NDRG1 were also observed in this investigation that accompanied PKCα stabilization (Fig. 9). Thus, NDRG1 demonstrates multi-modal mechanisms in suppressing WNT/β-catenin signaling in PC cells. Yet other mechanisms that mediate the activity of NDRG1 have been reported examining Huh7 and HepG2 hepatocellular carcinoma cells, where direct binding of NDRG1 to GSK-3β prevented its binding to β-catenin (90). This later effect of NDRG1 inhibited GSK-3β-mediated phosphorylation of β-catenin at Ser33, Ser37, and Thr41 (90).

Both the present investigation examining PC cells and our previous studies using prostate cancer and colon cancer cells (13) also demonstrated that NDRG1 expression inhibited β-catenin phosphorylation at Ser552 promoting its transcriptional activity (46). It has been additionally shown that activated AKT can phosphorylate GSK-3β at Ser9 (64). However, after NDRG1 overexpression, AKT activation is suppressed, with decreased p-AKT (Ser473) levels being observed. As such, the increased phosphorylation of GSK-3β (Ser9) after NDRG1 expression is consistent with an AKT-independent mechanism. There is also considerable crosstalk between AKT and PKCα in endometrial cancer, with AKT activation being negatively regulated by PKCα that had an anti-oncogenic effect (64). Similarly, in PC cells, *PKCα* silencing increased p-AKT (Ser473) and p-β-catenin (Ser552) levels that were consistent with the increase of PKCα after NDRG1 overexpression decreasing p-AKT (Ser473) and p-β-catenin (Ser552; Fig. 9).

Using neural cells (91) and platelets (63), studies implementing chemical inhibitors provided evidence that PKCα phosphorylates GSK-3β at Ser9 inhibiting its activity. This observation suggested the NDRG1-mediated upregulation of PKCα observed in PANC-1 cells after NDRG1 overexpression could be responsible for the increase in GSK-3β phosphorylation at Ser9 (Fig. 1*A*). However, *PKCα* silencing had no effect on GSK-3β Ser9 phosphorylation in PANC-1 cells, or paradoxically increased it in AsPC-1 cells, suggesting PKCα was not responsible for GSK-3β phosphorylation at Ser9 under these conditions.

PC remains a belligerent, leading cause of cancer-related death that has unfortunately doubled worldwide in the past 25 years (92). Current chemotherapies are woefully suboptimal, leading to an overall 5-year relative survival rate of approximately 10% (93). New therapeutics with a different mechanism of action are desperately needed. Metastasis is the major killer in cancer leading to 90% of deaths (72, 73). As such, there is an urgent need to identify novel targets and use that knowledge to specifically design and develop bespoke anti-metastatic drugs, as none exist in the clinics (94, 95). The metastasis suppressor, NDRG1, has recently become recognized as an important pharmacological target (96, 97). Our previous studies designed and synthesized novel thiosemicarbazone anti-cancer drugs that induce NDRG1 expression and potently inhibit primary tumor growth *in vivo* in a variety of cancers including several PC models (16, 34, 37, 38, 39, 40, 41, 42, 98).

The high potential of the thiosemicarbazones led to a Phase I clinical trial of our thiosemicarbazone anti-cancer drug, DpC (99, 100), and the subsequent synthesis of a new generation of analogs, including PPP44mT, that are patented and undergoing development (33, 74, 101). The mechanism of action of NDRG1 is mediated through the inhibition of multiple oncogenic pathways, including WNT signaling (16, 17, 47, 102). The ability of these thiosemicarbazones to upregulate NDRG1 expression has been demonstrated by our laboratory and others to (1) inhibit oncogenic WNT signaling; (2) decrease the nuclear translocation of β-catenin; and (3) inhibit the initial first step of metastasis, the epithelial–mesenchymal transition (7). Of note, the marked and selectively anti-proliferative and/or anti-metastatic activity of Dp44mT, DpC, and PPP44mT have been well characterized *in vitro* and/or *in vivo* (16, 33, 34, 38, 39, 41, 74).

The current investigation dissects novel molecular mechanisms involved in the inhibition of WNT signaling with our studies demonstrating that Dp44mT, DpC, and PPP44mT mimic genetically overexpressed NDRG1 by upregulating PKCα, downregulating β-catenin and PAK4, inhibiting GSK-3β expression and activation, and suppressing the expression of a key WNT effector, cyclin D1. Targeting WNT signaling has been described as a potential therapeutic strategy for PC (30), with the novel molecular mechanisms dissected herein not being observed in other clinically used pharmacopoeia. As such, the development of these thiosemicarbazones as effective anti-cancer agents warrants further vigorous investigation.

Of interest, the upregulation of total PKCα protein and downregulation of total β-catenin by the NDRG1-inducing therapeutics, DpC and DFO, was also demonstrated in SK-Mel-28 melanoma cells (Fig. S2*A*). Furthermore, while the expression of PKCα total protein was demonstrated to markedly vary between tumor cell-types in culture, *NDRG1* silencing resulted in the downregulation of PKCα protein levels in SK-Mel-28 melanoma cells, AsPC-1 pancreatic cancer cells (positive control), T47D breast cancer cells, Kelly and SH-SY5Y neuroblastoma cells (Fig. S2*B*). These studies suggest the anti-tumor activity of our NDRG1-inducing agents (16, 34, 37, 38, 39, 40, 41, 42, 98) and NDRG1 itself against multiple cancer-types (1, 2, 3, 4), could be mediated, at least in part, *via* its effect on PKCα expression.

In conclusion, for the first time, the current investigation reports a novel mechanism of NDRG1-mediated antagonism of WNT signaling in PC cells facilitated by a novel metabolon involving the association of NDRG1, PKCα, and β-catenin that may effectively promote the activity of these proteins (Fig. 9). In fact, an association of NDRG1 with PKCα was demonstrated that may lead to the observed stabilization of PKCα and its increased protein levels. The upregulation of PKCα mediated the destabilizing phosphorylation of β-catenin at Ser33, Ser37, and Thr41, and a marked decrease in its expression (Fig. 9). This effect of NDRG1 expression was accompanied by the downregulation of PAK4 that inhibits β-catenin nuclear translocation. Finally, NDRG1 overexpression upregulates PKCα that suppresses AKT phosphorylation at Ser473, and the phosphorylation of β-catenin at Ser552 that promotes its transcriptional activity (Fig. 9). Dissection of these mechanisms is important in understanding the potent pharmacological inhibition of PC growth by innovative pharmacopeia of the thiosemicarbazone class that are under development as anti-cancer drugs (16, 18, 34).

## Experimental procedures

### Cell culture

Human PANC-1 and AsPC-1 PC cells, SK-Mel-28 melanoma cells, T47D breast cancer cells, MCF-7 breast cancer cells, Kelly neuroblastoma cells, and SH-SY5Y neuroblastoma cells, were purchased from the American Type Culture Collection (ATCC, Manassas, VA). Cell-types were authenticated based on viability, recovery, growth, morphology, and cytogenetic analysis (*i.e.*, antigen expression, DNA profile, and isoenzymology) by the supplier. The empty pCMV-tag2-FLAG plasmid or this plasmid containing the *NDRG1* open reading frame (pCMV-tag2-FLAG-NDRG1) were stably transfected into PANC-1 cells to generate PANC-1-vector control (VC) cells and NDRG1 overexpressing (NDRG1) cells, respectively (9).

PANC-1 cells were grown using Dulbecco's Modified Eagle Medium (DMEM) containing 10% (v/v) fetal bovine serum (FBS), 1% non-essential amino acids (Cat.#: M7145, Sigma Aldrich), 1% streptomycin/penicillin/glutamine (Cat.#: G1146, Sigma Aldrich) and 1% sodium pyruvate (Cat.#: S8636, Sigma Aldrich). PANC-1 VC and NDRG1 cells were also grown and maintained in the same medium with 0.4 mg/ml G418 (Geneticin; Thermo Fisher Scientific) to select for cells transfected with the plasmid. AsPC-1 pancreatic cancer cells, SH-SY5Y and KELLY neuroblastoma cells were grown in Roswell Park Memorial Institute (RPMI) media, which included the same supplements listed above for DMEM. T47D breast cancer cells were grown in DMEM media with the supplements above. SK-Mel-28 melanoma cells, MCF-7 breast cancer cells were grown in Eagle’s modified minimum essential media (MEM), with the supplements above. All cells were grown in an incubator at 37 °C with an atmosphere of 5% CO_2_/95% air. In experiments studying the effect of WNT3a ligand, human WNT3a (Cat#: H17001, Sigma Aldrich); was reconstituted to 100 μg/ml in PBS containing 0.1% endotoxin-free human serum albumin. PANC-1 VC and NDRG1 cells were incubated with or without WNT3a (100 ng/ml)-containing DMEM media for 24 h/37 °C.

### Genetic silencing of *NDRG1* and *PKCα via* small interfering RNA (siRNA)

A non-targeting negative control siRNA (siControl; Cat.#: 4390843, Thermo Fisher Scientific), and siRNA specific for *NDRG1* (siNDRG1; Cat.#: 4392420; Invitrogen, Carlsbad, CA) or *PKCα* (*PRKCA;* siPKCα; Cat.# 4390824; Assay ID: s11094; Invitrogen) were used. Sequences of each siRNA are available online from the suppliers. The siRNAs were reverse transiently transfected into PANC-1 cells using Lipofectamine RNAiMAXR (Cat.#: 13778100; Invitrogen) and incubated for 48 h/37 °C, respectively. Then total cellular protein extraction and western blotting was performed, as described below.

### Protein extraction

Following the removal of the cells from the culture plates, the suspensions were disrupted on ice using a sonicator 150 (Branson, MO), followed by centrifugation at 13,200 rpm/40 min/4 °C using a Fresco 17 centrifuge (Thermo Fisher Scientific). Subsequently, the supernatant was separated from the pellet, and the protein concentration determined using the bicinchoninic acid (BCA) protein assay kit (Thermo Fisher Scientific) using a UV-Vis spectrophotometer (Shimadzu).

### SDS-PAGE and Western blot analysis

Protein lysates were prepared after the addition of β-mercaptoethanol (Sigma-Aldrich) and then heated for 5 min/95 °C on a heating block (103). SDS-PAGE (8, 10, or 12% gels) was then performed and the separated proteins were transferred for 16 h onto a PVDF membrane (0.45 μM pore size; Millipore) at 30 V/4 °C. Once the transfer was complete, the membrane was soaked in 100% methanol for 30 s and dried at 37 °C/2 h.

Membranes were blocked in 5% skim milk or bovine serum albumin (BSA; Cat.#: A9418 Sigma-Aldrich) solution in TBS-T at room temperature for 1.5 h. Primary antibodies were diluted in 5% skim milk or BSA in TBS-T and incubated with the appropriate membranes overnight at 4 °C. The membranes were then incubated with secondary antibody diluted in 5% skim milk at room temperature for 1 h, followed by washing in TBS-T (3 times/5 min). A list of primary and secondary antibodies including catalogue numbers are provided in Table S1. The specificity of each antibody was validated by the suppliers and supported by the assessment of the molecular weight of each protein detected (using molecular weight markers).

The antibody-antigen complex was detected after incubation with Western HRP substrate (Cat no. WBLUF0500: Millipore) for 1 min. The signal produced was detected and imaged with a Sapphire Biomolecular Imager (Azure Biosystems, CA). Signals were normalized to the total protein loaded to each lane (80 μg/lane), with equal loading being determined by reference to the house keeping protein, β-actin (Table S1). For every Western blot performed, β-actin was also probed to ensure appropriate normalization of protein loading.

### Co-immunoprecipitation (co-IP)

Protein extraction from cells was performed using immunoprecipitation lysis buffer (Pierce IP Lysis Buffer, Thermo Fisher Scientific). Standard methods previously used in our laboratory were utilized for co-immunoprecipitation (co-IP) (66). Lysate protein concentration was determined using the BCA protein assay described above. Then, 400 μg of protein was incubated overnight with either the primary antibody specific to the target protein (Table S1) or a respective isotype control antibody (Isotype Con. Ab; Cell Signaling Technology; Table S1) used to assess the non-specific binding of immunoglobulins to the protein of interest.

Magnetic bead solution (30 mg/ml; Pierce Protein A/G Magnetic Beads; Thermo Fisher Scientific) was blocked using 5% BSA/PBS solution overnight and added to the antibody: lysate solution and incubated for 1.5 h/4 °C. The samples containing the magnetic beads and antibody: lysate solution were then placed on a magnet and the supernatant was discarded. The precipitate was washed 5 times with ice-cold PBS to remove non-specifically bound proteins on the magnetic beads. Then, the magnetic beads were suspended in 5× loading dye containing β-mercaptoethanol (Sigma-Aldrich) solution, and the bound proteins denatured by boiling at 5 min/95 °C. This solution was used for SDS-PAGE and western blotting. For comparison, input samples containing 80 μg of protein were run simultaneously using SDS-PAGE implementing the western blotting protocol above.

### Cycloheximide chase studies

The half-lives of NDRG1 and PKCα were evaluated using a cycloheximide (CHX) chase assay implementing previously established methods (17). PANC-1 VC and NDRG1 cells were seeded and incubated overnight at 37 °C and the cells were preincubated for 1 h/37 °C with the protein synthesis inhibitor, CHX (15 μg/ml; Sigma-Aldrich). A chase was then performed for 0, 2, 4, 8, 10, 12, and 14 h/37 °C in the presence of CHX (15 μg/ml). Subsequently, the cells were lysed, and protein extracted for SDS-PAGE and Western blotting.

### Confocal microscopy

Initially, PANC-1 VC and NDRG1 cells (400,000 cells/condition) were seeded on sterile coverslips and incubated for 24 h/37 °C in media supplemented with serum or WNT3a ligand (100 ng/ml). Fixation was then performed by implementing a 10 min incubation with 4% (w/v) paraformaldehyde/PBS/20 °C, followed by cell permeabilization using 0.1% Triton X-100/PBS using an incubation for 10 min/20 °C. Following permeabilization, fixed cells were washed again, and the coverslips were blocked for 1 h with 5% BSA/0.3 M glycine in PBS/20 °C. Then, primary antibodies were diluted in 1% BSA/PBS, added to each well, and incubated overnight at 4 °C on an orbital shaker at 200 rpm.

After overnight incubation with the primary antibodies, the wells were washed three times with 1% BSA/PBS. Secondary antibody solutions were prepared to the desired concentration in 1% BSA/PBS and added to all wells, followed by incubation at room temperature for 1 h on an orbital shaker at 200 rpm. Coverslips were subsequently washed and mounted onto a slide using ProLong Gold DAPI mounting solution (Cat. # P36935; Invitrogen). The slides were then left to dry in the dark at room temperature for a minimum of 6 h. An Olympus FV3000RS confocal microscope (Evident Scientific) was used with a 60× and 100× objective to visualize cells. The images of visualized cells were then examined using Olympus Fluoview software, and in some studies, images were processed for deconvolution analysis with CellSens software (Olympus) to improve contrast and image resolution. Fluorescence intensities and co-localization analysis were quantified using ImageJ software (NIHD). The Pearson correlation coefficient (*r*) was calculated using the Image J plugin, JACoP. All primary and secondary antibodies used for confocal microscopy are listed in Table S1.

In order to maintain uniformity samples for the accurate assessment of colocalization, the following measures were implemented during sample preparation and the image acquisition process; (1) A consistent number of cells were seeded on each coverslip per sample to minimize variation in sample thickness and to ensure the presence of only a monolayer of cells; (2) images of each channels per sample were acquired at the same XYZ plane (relative to other channels); (3) within each experiment all microscope acquisition parameters (*e.g.*, pinhole aperture settings) were kept constant.

The primary and secondary antibodies for immunostaining in the confocal analysis were validated by the manufacturers to be suitable for immunofluorescence imaging. In addition, the following measures were employed to further validate the antibodies: (1) Utilizing negative controls, such as omitting the primary antibodies (2) employing isotype controls (*i.e.*, implementing isotype control antibodies); with matching concentration to the primary antibody of interest; and (3) adding positive controls (*i.e.*, validated cell-types that possess the molecule of interest overexpressed and/or silenced).

### Statistical analysis

Results for all experiments were generated from three independent experiments and presented as mean ± standard deviation. Data were normally distributed around the mean with a two-tailed Student’s paired *t* test being used for statistical analysis implementing GraphPad Prism 9.2.0 software. Data were considered statistically significant when *p* < 0.05.

## Data availability

The datasets used and/or analyzed during the current study are available from the corresponding author on request.

## Supporting information

This article contains supporting information.

## Conflict of interest

The authors declare that they have no known competing financial interests or personal relationships that could have appeared to influence the work reported in this paper.

## References

[1] Bandyopadhyay S., Pai S.K., Gross S.C., Hirota S., Hosobe S., Miura K. (2003). The Drg-1 gene suppresses tumor metastasis in prostate cancer. Cancer Res..

[2] Maruyama Y., Ono M., Kawahara A., Yokoyama T., Basaki Y., Kage M. (2006). Tumor growth suppression in pancreatic cancer by a putative metastasis suppressor gene Cap43/NDRG1/Drg-1 through modulation of angiogenesis. Cancer Res..

[3] Strzelczyk B., Szulc A., Rzepko R., Kitowska A., Skokowski J., Szutowicz A. (2009). Identification of high-risk stage II colorectal tumors by combined analysis of the NDRG1 gene expression and the depth of tumor invasion. Ann. Surg. Oncol..

[4] Deng Z., Richardson D.R. (2023). The myc family and the metastasis suppressor NDRG1: targeting key molecular interactions with innovative therapeutics. Pharmacol. Rev..

[5] Fang B.A., Kovačević Ž., Park K.C., Kalinowski D.S., Jansson P.J., Lane D.J. (2014). Molecular functions of the iron-regulated metastasis suppressor, NDRG1, and its potential as a molecular target for cancer therapy. Biochim. Biophys. Acta.

[6] Bae D.H., Jansson P.J., Huang M.L., Kovacevic Z., Kalinowski D., Lee C.S. (2013). The role of NDRG1 in the pathology and potential treatment of human cancers. J. Clin. Pathol..

[7] Chen Z., Zhang D., Yue F., Zheng M., Kovacevic Z., Richardson D.R. (2012). The iron chelators Dp44mT and DFO inhibit TGF-β-induced epithelial-mesenchymal transition via up-regulation of N-Myc downstream-regulated gene 1 (NDRG1). J. Biol. Chem..

[8] Hosoi F., Izumi H., Kawahara A., Murakami Y., Kinoshita H., Kage M. (2009). N-myc downstream regulated gene 1/Cap43 suppresses tumor growth and angiogenesis of pancreatic cancer through attenuation of inhibitor of kappaB kinase beta expression. Cancer Res..

[9] Kovacevic Z., Chikhani S., Lui G.Y., Sivagurunathan S., Richardson D.R. (2013). The iron-regulated metastasis suppressor NDRG1 targets NEDD4L, PTEN, and SMAD4 and inhibits the PI3K and Ras signaling pathways. Antioxid. Redox Signal..

[10] Lane D.J., Mills T.M., Shafie N.H., Merlot A.M., Saleh Moussa R., Kalinowski D.S. (2014). Expanding horizons in iron chelation and the treatment of cancer: role of iron in the regulation of ER stress and the epithelial-mesenchymal transition. Biochim. Biophys. Acta.

[11] Shi X.H., Larkin J.C., Chen B., Sadovsky Y. (2013). The expression and localization of N-myc downstream-regulated gene 1 in human trophoblasts. PLoS One.

[12] Park K.C., Menezes S.V., Kalinowski D.S., Sahni S., Jansson P.J., Kovacevic Z. (2018). Identification of differential phosphorylation and sub-cellular localization of the metastasis suppressor, NDRG1. Biochim. Biophys. Acta Mol. Basis Dis..

[13] Jin R., Liu W., Menezes S., Yue F., Zheng M., Kovacevic Z. (2014). The metastasis suppressor NDRG1 modulates the phosphorylation and nuclear translocation of β-catenin through mechanisms involving FRAT1 and PAK4. J. Cell Sci..

[14] Sun J., Zhang D., Zheng Y., Zhao Q., Zheng M., Kovacevic Z. (2013). Targeting the metastasis suppressor, NDRG1, using novel iron chelators: regulation of stress fiber-mediated tumor cell migration via modulation of the ROCK1/pMLC2 signaling pathway. Mol. Pharmacol..

[15] Dixon K.M., Lui G.Y., Kovacevic Z., Zhang D., Yao M., Chen Z. (2013). Dp44mT targets the AKT, TGF-beta and ERK pathways via the metastasis suppressor NDRG1 in normal prostate epithelial cells and prostate cancer cells. Br. J. Cancer.

[16] Kovacevic Z., Menezes S.V., Sahni S., Kalinowski D.S., Bae D.H., Lane D.J. (2016). The metastasis suppressor, N-myc downstream-regulated gene-1 (NDRG1), down-regulates the ErbB family of receptors to inhibit downstream oncogenic signaling pathways. J. Biol. Chem..

[17] Menezes S.V., Kovacevic Z., Richardson D.R. (2019). The metastasis suppressor NDRG1 down-regulates the epidermal growth factor receptor via a lysosomal mechanism by up-regulating mitogen-inducible gene 6. J. Biol. Chem..

[18] Geleta B., Park K.C., Jansson P.J., Sahni S., Maleki S., Xu Z. (2021). Breaking the cycle: targeting of NDRG1 to inhibit bi-directional oncogenic cross-talk between pancreatic cancer and stroma. FASEB J..

[19] Park K.C., Geleta B., Leck L.Y.W., Paluncic J., Chiang S., Jansson P.J. (2020). Thiosemicarbazones suppress expression of the c-Met oncogene by mechanisms involving lysosomal degradation and intracellular shedding. J. Biol. Chem..

[20] Domoto T., Uehara M., Bolidong D., Minamoto T. (2020). Glycogen synthase kinase 3β in cancer biology and treatment. Cells.

[21] Clevers H., Nusse R. (2012). Wnt/beta-catenin signaling and disease. Cell.

[22] Zeng G., Germinaro M., Micsenyi A., Monga N.K., Bell A., Sood A. (2006). Aberrant Wnt/beta-catenin signaling in pancreatic adenocarcinoma. Neoplasia.

[23] Aguilera K.Y., Dawson D.W. (2021). Wnt ligand dependencies in pancreatic cancer. Front. Cell Dev. Biol..

[24] Nakashima S. (2002). Protein kinase C alpha (PKC alpha): regulation and biological function. J. Biochem..

[25] Clemens M.J., Trayner I., Menaya J. (1992). The role of protein kinase C isoenzymes in the regulation of cell proliferation and differentiation. J. Cell Sci..

[26] Shin S.Y., Kim C.G., Jho E.H., Rho M.S., Kim Y.S., Kim Y.H. (2004). Hydrogen peroxide negatively modulates Wnt signaling through downregulation of beta-catenin. Cancer Lett..

[27] Gwak J., Yoo Y.S., Choi Y.J., Oh S. (2014). Interaction of PKCα with the armadillo repeats facilitates the N-terminal phosphorylation of β-catenin. Biochem. Biophys. Res. Commun..

[28] Ferlay J., Soerjomataram I., Dikshit R., Eser S., Mathers C., Rebelo M. (2015). Cancer incidence and mortality worldwide: sources, methods and major patterns in GLOBOCAN 2012. Int. J. Cancer.

[29] Hidalgo M., Cascinu S., Kleeff J., Labianca R., Lohr J.M., Neoptolemos J. (2015). Addressing the challenges of pancreatic cancer: future directions for improving outcomes. Pancreatology.

[30] Javadinia S.A., Shahidsales S., Fanipakdel A., Joudi-Mashhad M., Mehramiz M., Talebian S. (2019). Therapeutic potential of targeting the Wnt/beta-catenin pathway in the treatment of pancreatic cancer. J. Cell. Biochem..

[31] Liu W., Xing F., Iiizumi-Gairani M., Okuda H., Watabe M., Pai S.K. (2012). N-myc downstream regulated gene 1 modulates Wnt-β-catenin signalling and pleiotropically suppresses metastasis. EMBO Mol. Med..

[32] Shi X., Cen Y., Shan L., Tian L., Zhu E., Yuan H. (2022). N-myc downstream regulated gene 1 suppresses osteoblast differentiation through inactivating Wnt/beta-catenin signaling. Stem Cell. Res. Ther..

[33] Wijesinghe T.P., Kaya B., Gonzalvez M.A., Harmer J.R., Gholam Azad M., Bernhardt P.V. (2023). Steric blockade of oxy-myoglobin oxidation by thiosemicarbazones: structure-activity relationships of the novel PPP4pT series. J. Med. Chem..

[34] Kovacevic Z., Chikhani S., Lovejoy D.B., Richardson D.R. (2011). Novel thiosemicarbazone iron chelators induce up-regulation and phosphorylation of the metastasis suppressor N-myc down-stream regulated gene 1: a new strategy for the treatment of pancreatic cancer. Mol. Pharmacol..

[35] Geleta B., Tout F.S., Lim S.C., Sahni S., Jansson P.J., Apte M.V. (2022). Targeting Wnt/tenascin C-mediated cross talk between pancreatic cancer cells and stellate cells via activation of the metastasis suppressor NDRG1. J. Biol. Chem..

[36] Dharmasivam M., Kaya B., Wijesinghe T.P., Richardson V., Harmer J.R., Gonzalvez M.A. (2024). Differential transmetallation of complexes of the anti-cancer thiosemicarbazone, Dp4e4mT: effects on anti-proliferative efficacy, redox activity, oxy-myoglobin and oxy-hemoglobin oxidation. Chem. Sci..

[37] Yuan J., Lovejoy D.B., Richardson D.R. (2004). Novel di-2-pyridyl-derived iron chelators with marked and selective antitumor activity: in vitro and in vivo assessment. Blood.

[38] Whitnall M., Howard J., Ponka P., Richardson D.R. (2006). A class of iron chelators with a wide spectrum of potent antitumor activity that overcomes resistance to chemotherapeutics. Proc. Natl. Acad. Sci. U. S. A..

[39] Guo Z.L., Richardson D.R., Kalinowski D.S., Kovacevic Z., Tan-Un K.C., Chan G.C. (2016). The novel thiosemicarbazone, di-2-pyridylketone 4-cyclohexyl-4-methyl-3-thiosemicarbazone (DpC), inhibits neuroblastoma growth in vitro and in vivo via multiple mechanisms. J. Hematol. Oncol..

[40] Lim S.C., Jansson P.J., Assinder S.J., Maleki S., Richardson D.R., Kovacevic Z. (2020). Unique targeting of androgen-dependent and -independent AR signaling in prostate cancer to overcome androgen resistance. FASEB J..

[41] Shehadeh-Tout F., Milioli H.H., Roslan S., Jansson P.J., Dharmasivam M., Graham D. (2023). Innovative thiosemicarbazones that induce multi-modal mechanisms to down-regulate estrogen-, progesterone-, androgen- and prolactin-receptors in breast cancer. Pharmacol. Res..

[42] Yu Y., Suryo Rahmanto Y., Richardson D.R. (2012). Bp44mT: an orally active iron chelator of the thiosemicarbazone class with potent anti-tumour efficacy. Br. J. Pharmacol..

[43] Sun J., Zhang D., Bae D.H., Sahni S., Jansson P., Zheng Y. (2013). Metastasis suppressor, NDRG1, mediates its activity through signaling pathways and molecular motors. Carcinogenesis.

[44] Menezes S.V., Sahni S., Kovacevic Z., Richardson D.R. (2017). Interplay of the iron-regulated metastasis suppressor NDRG1 with epidermal growth factor receptor (EGFR) and oncogenic signaling. J. Biol. Chem..

[45] Zhao X., Richardson D.R. (2023). The role of the NDRG1 in the pathogenesis and treatment of breast cancer. Biochim. Biophys. Acta Rev. Cancer.

[46] Fang D., Hawke D., Zheng Y., Xia Y., Meisenhelder J., Nika H. (2007). Phosphorylation of beta-catenin by AKT promotes beta-catenin transcriptional activity. J. Biol. Chem..

[47] Menezes S.V., Fouani L., Huang M.L.H., Geleta B., Maleki S., Richardson A. (2019). The metastasis suppressor, NDRG1, attenuates oncogenic TGF-beta and NF-kappaB signaling to enhance membrane E-cadherin expression in pancreatic cancer cells. Carcinogenesis.

[48] Ghalayini M.K., Dong Q., Richardson D.R., Assinder S.J. (2013). Proteolytic cleavage and truncation of NDRG1 in human prostate cancer cells, but not normal prostate epithelial cells. Biosci. Rep..

[49] Sahni S., Park K.C., Kovacevic Z., Richardson D.R. (2019). Two mechanisms involving the autophagic and proteasomal pathways process the metastasis suppressor protein, N-myc downstream regulated gene 1. Biochim. Biophys. Acta Mol. Basis Dis..

[50] Aberle H., Bauer A., Stappert J., Kispert A., Kemler R. (1997). beta-catenin is a target for the ubiquitin-proteasome pathway. EMBO J..

[51] Cross D.A., Alessi D.R., Cohen P., Andjelkovich M., Hemmings B.A. (1995). Inhibition of glycogen synthase kinase-3 by insulin mediated by protein kinase B. Nature.

[52] Alessi D.R., Deak M., Casamayor A., Caudwell F.B., Morrice N., Norman D.G. (1997). 3-Phosphoinositide-dependent protein kinase-1 (PDK1): structural and functional homology with the Drosophila DSTPK61 kinase. Curr. Biol..

[53] Li Y., Shao Y., Tong Y., Shen T., Zhang J., Li Y. (2012). Nucleo-cytoplasmic shuttling of PAK4 modulates beta-catenin intracellular translocation and signaling. Biochim. Biophys. Acta.

[54] Dart A.E., Wells C.M. (2013). P21-activated kinase 4--not just one of the PAK. Eur. J. Cell Biol..

[55] Rubinfeld B., Albert I., Porfiri E., Fiol C., Munemitsu S., Polakis P. (1996). Binding of GSK3beta to the APC-beta-catenin complex and regulation of complex assembly. Science.

[56] Fang X., Yu S.X., Lu Y., Bast R.C., Woodgett J.R., Mills G.B. (2000). Phosphorylation and inactivation of glycogen synthase kinase 3 by protein kinase A. Proc. Natl. Acad. Sci. U. S. A..

[57] Wu D., Pan W. (2010). GSK3: a multifaceted kinase in Wnt signaling. Trends Biochem. Sci..

[58] He L., Fei D.L., Nagiec M.J., Mutvei A.P., Lamprakis A., Kim B.Y. (2019). Regulation of GSK3 cellular location by FRAT modulates mTORC1-dependent cell growth and sensitivity to rapamycin. Proc. Natl. Acad. Sci. U. S. A..

[59] Nusse R., Clevers H. (2017). Wnt/beta-catenin signaling, disease, and emerging therapeutic modalities. Cell.

[60] Hsu A.H., Lum M.A., Shim K.S., Frederick P.J., Morrison C.D., Chen B. (2018). Crosstalk between PKCalpha and PI3K/AKT signaling is tumor suppressive in the endometrium. Cell Rep..

[61] Krishnankutty A., Kimura T., Saito T., Aoyagi K., Asada A., Takahashi S.I. (2017). In vivo regulation of glycogen synthase kinase 3beta activity in neurons and brains. Sci. Rep..

[62] Hughes K., Nikolakaki E., Plyte S.E., Totty N.F., Woodgett J.R. (1993). Modulation of the glycogen synthase kinase-3 family by tyrosine phosphorylation. EMBO J..

[63] Moore S.F., van den Bosch M.T., Hunter R.W., Sakamoto K., Poole A.W., Hers I. (2013). Dual regulation of glycogen synthase kinase 3 (GSK3)α/β by protein kinase C (PKC)α and Akt promotes thrombin-mediated integrin αIIbβ3 activation and granule secretion in platelets. J. Biol. Chem..

[64] Li L., Sampat K., Hu N., Zakari J., Yuspa S.H. (2006). Protein kinase C negatively regulates Akt activity and modifies UVC-induced apoptosis in mouse keratinocytes. J. Biol. Chem..

[65] Weiler M., Blaes J., Pusch S., Sahm F., Czabanka M., Luger S. (2014). mTOR target NDRG1 confers MGMT-dependent resistance to alkylating chemotherapy. Proc. Natl. Acad. Sci. U. S. A..

[66] Lim S., Geleta B., Maleki S., Richardson D.R., Kovačević Z. (2021). The metastasis suppressor NDRG1 directly regulates androgen receptor signaling in prostate cancer. J. Biol. Chem..

[67] Dunn K.W., Kamocka M.M., McDonald J.H. (2011). A practical guide to evaluating colocalization in biological microscopy. Am. J. Physiol. Cell Physiol..

[68] Oloumi A., Syam S., Dedhar S. (2006). Modulation of Wnt3a-mediated nuclear beta-catenin accumulation and activation by integrin-linked kinase in mammalian cells. Oncogene.

[69] Lampasso J.D., Marzec N., Margarone J., Dziak R. (2002). Role of protein kinase C alpha in primary human osteoblast proliferation. J. Bone Miner. Res..

[70] Gruber J.R., Desai S., Blusztajn J.K., Niles R.M. (1995). Retinoic acid specifically increases nuclear PKC alpha and stimulates AP-1 transcriptional activity in B16 mouse melanoma cells. Exp. Cell Res..

[71] Ai R., Sun Y., Guo Z., Wei W., Zhou L., Liu F. (2016). NDRG1 overexpression promotes the progression of esophageal squamous cell carcinoma through modulating Wnt signaling pathway. Cancer Biol. Ther..

[72] Seyfried T.N., Huysentruyt L.C. (2013). On the origin of cancer metastasis. Crit. Rev. Oncog..

[73] Chaffer C.L., Weinberg R.A. (2011). A perspective on cancer cell metastasis. Science.

[74] Dharmasivam M., Kaya B., Wijesinghe T., Gholam Azad M., Gonzalvez M.A., Hussaini M. (2023). Designing tailored thiosemicarbazones with bespoke properties: the styrene moiety imparts potent activity, inhibits heme center oxidation, and results in a novel “Stealth Zinc(II) complex”. J. Med. Chem..

[75] Le N.T., Richardson D.R. (2004). Iron chelators with high antiproliferative activity up-regulate the expression of a growth inhibitory and metastasis suppressor gene: a link between iron metabolism and proliferation. Blood.

[76] Stacy A.E., Palanimuthu D., Bernhardt P.V., Kalinowski D.S., Jansson P.J., Richardson D.R. (2016). Structure-activity relationships of di-2-pyridylketone, 2-benzoylpyridine, and 2-acetylpyridine thiosemicarbazones for overcoming pgp-mediated drug resistance. J. Med. Chem..

[77] Kalinowski D.S., Richardson D.R. (2005). The evolution of iron chelators for the treatment of iron overload disease and cancer. Pharmacol. Rev..

[78] Richardson D.R., Ponka P. (1998). Development of iron chelators to treat iron overload disease and their use as experimental tools to probe intracellular iron metabolism. Am. J. Hematol..

[79] Richardson D., Ponka P., Baker E. (1994). The effect of the iron(III) chelator, desferrioxamine, on iron and transferrin uptake by the human malignant melanoma cell. Cancer Res..

[80] Wong M.C.S., Jiang J.Y., Liang M., Fang Y., Yeung M.S., Sung J.J.Y. (2017). Global temporal patterns of pancreatic cancer and association with socioeconomic development. Sci. Rep..

[81] Sugiki T., Taketomi Y., Kikuchi-Yanoshita R., Murakami M., Kudo I. (2004). Association of N-myc downregulated gene 1 with heat-shock cognate protein 70 in mast cells. Biol. Pharm. Bull..

[82] Banz V.M., Medova M., Keogh A., Furer C., Zimmer Y., Candinas D. (2009). Hsp90 transcriptionally and post-translationally regulates the expression of NDRG1 and maintains the stability of its modifying kinase GSK3beta. Biochim. Biophys. Acta.

[83] Yanatori I., Richardson D.R., Toyokuni S., Kishi F. (2017). The iron chaperone poly(rC)-binding protein 2 forms a metabolon with the heme oxygenase 1/cytochrome P450 reductase complex for heme catabolism and iron transfer. J. Biol. Chem..

[84] Bulutoglu B., Garcia K.E., Wu F., Minteer S.D., Banta S. (2016). Direct evidence for metabolon formation and substrate channeling in recombinant TCA cycle enzymes. ACS Chem. Biol..

[85] Omini J., Wojciechowska I., Skirycz A., Moriyama H., Obata T. (2021). Association of the malate dehydrogenase-citrate synthase metabolon is modulated by intermediates of the Krebs tricarboxylic acid cycle. Sci. Rep..

[86] Wagner A. (2001). The yeast protein interaction network evolves rapidly and contains few redundant duplicate genes. Mol. Biol. Evol..

[87] Dixit P.D., Maslov S. (2013). Evolutionary capacitance and control of protein stability in protein-protein interaction networks. PLoS Comput. Biol..

[88] Jeong H., Mason S.P., Barabasi A.L., Oltvai Z.N. (2001). Lethality and centrality in protein networks. Nature.

[89] Keranen L.M., Dutil E.M., Newton A.C. (1995). Protein kinase C is regulated in vivo by three functionally distinct phosphorylations. Curr. Biol..

[90] Lu W.J., Chua M.S., Wei W., So S.K. (2015). NDRG1 promotes growth of hepatocellular carcinoma cells by directly interacting with GSK-3β and Nur77 to prevent β-catenin degradation. Oncotarget.

[91] Kirshenboim N., Plotkin B., Shlomo S.B., Kaidanovich-Beilin O., Eldar-Finkelman H. (2004). Lithium-mediated phosphorylation of glycogen synthase kinase-3beta involves PI3 kinase-dependent activation of protein kinase C-alpha. J. Mol. Neurosci..

[92] Klein A.P. (2021). Pancreatic cancer epidemiology: understanding the role of lifestyle and inherited risk factors. Nat. Rev. Gastroenterol. Hepatol..

[93] Cai J., Chen H., Lu M., Zhang Y., Lu B., You L. (2021). Advances in the epidemiology of pancreatic cancer: trends, risk factors, screening, and prognosis. Cancer Lett..

[94] Anderson R.L., Balasas T., Callaghan J., Coombes R.C., Evans J., Hall J.A. (2019). A framework for the development of effective anti-metastatic agents. Nat. Rev. Clin. Oncol..

[95] Gandalovicova A., Rosel D., Fernandes M., Vesely P., Heneberg P., Cermak V. (2017). Migrastatics-anti-metastatic and anti-invasion drugs: promises and challenges. Trends Cancer.

[96] Park K.C., Paluncic J., Kovacevic Z., Richardson D.R. (2020). Pharmacological targeting and the diverse functions of the metastasis suppressor, NDRG1, in cancer. Free Radic. Biol. Med..

[97] Chekmarev J., Azad M.G., Richardson D.R. (2021). The oncogenic signaling disruptor, NDRG1: molecular and cellular mechanisms of activity. Cells.

[98] Lovejoy D.B., Sharp D.M., Seebacher N., Obeidy P., Prichard T., Stefani C. (2012). Novel second-generation di-2-pyridylketone thiosemicarbazones show synergism with standard chemotherapeutics and demonstrate potent activity against lung cancer xenografts after oral and intravenous administration in vivo. J. Med. Chem..

[99] Jansson P.J., Kalinowski D.S., Lane D.J., Kovacevic Z., Seebacher N.A., Fouani L. (2015). The renaissance of polypharmacology in the development of anti-cancer therapeutics: inhibition of the “Triad of Death” in cancer by di-2-pyridylketone thiosemicarbazones. Pharmacol. Res..

[100] Wijesinghe T.P., Dharmasivam M., Dai C.C., Richardson D.R. (2021). Innovative therapies for neuroblastoma: the surprisingly potent role of iron chelation in up-regulating metastasis and tumor suppressors and down-regulating the key oncogene, N-myc. Pharmacol. Res..

[101] Richardson D.R., Dharmasivam M. (2023). Anti-Cancer Compounds and Uses Thereof.

[102] Wangpu X., Lu J., Xi R., Yue F., Sahni S., Park K.C. (2016). Targeting the metastasis suppressor, N-myc downstream regulated gene-1, with novel di-2-pyridylketone thiosemicarbazones: suppression of tumor cell migration and cell-collagen adhesion by inhibiting focal adhesion kinase/paxillin signaling. Mol. Pharmacol..

[103] Gao J., Richardson D.R. (2001). The potential of iron chelators of the pyridoxal isonicotinoyl hydrazone class as effective antiproliferative agents, IV: the mechanisms involved in inhibiting cell-cycle progression. Blood.

