# Supplementary material for: Multi-modal mechanisms of the metastasis suppressor, NDRG1: Inhibition of WNT/β-catenin signaling by stabilization of protein kinase Cα
Source: J Biol Chem. 2024 May 28;300(7):107417. doi: 10.1016/j.jbc.2024.107417 (PMC11261793; doi:10.1016/j.jbc.2024.107417)
Supplement: Supporting information [file mmc1.docx]

**Supporting Information**

**
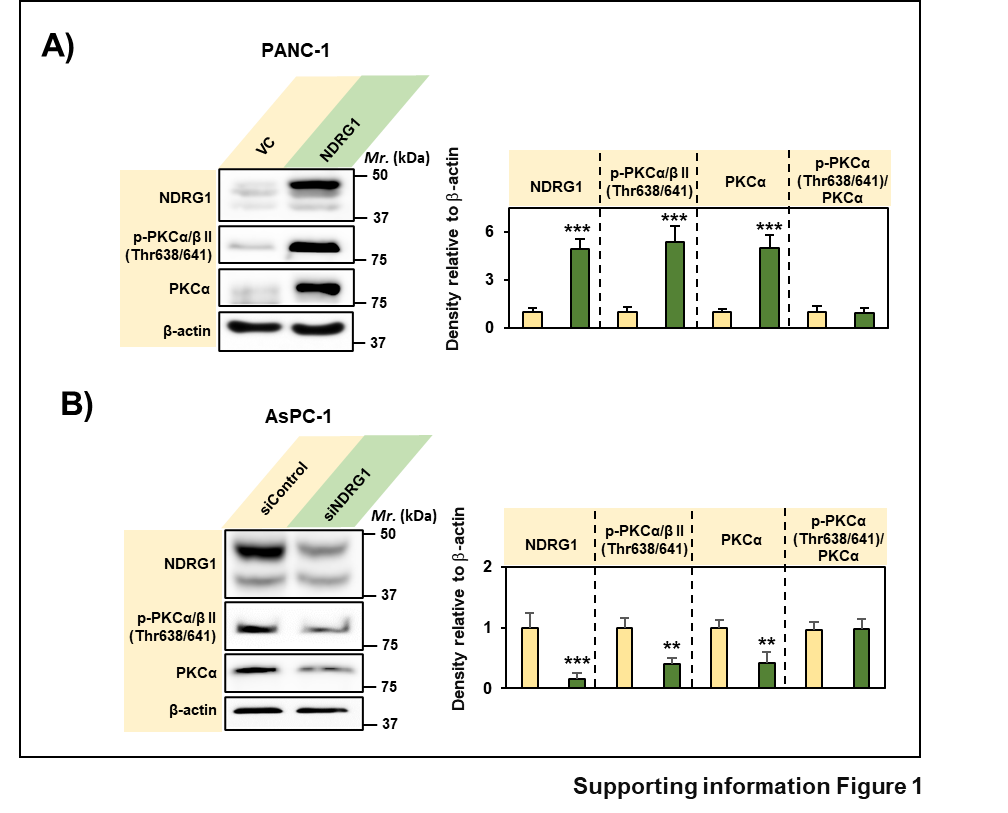
**

**
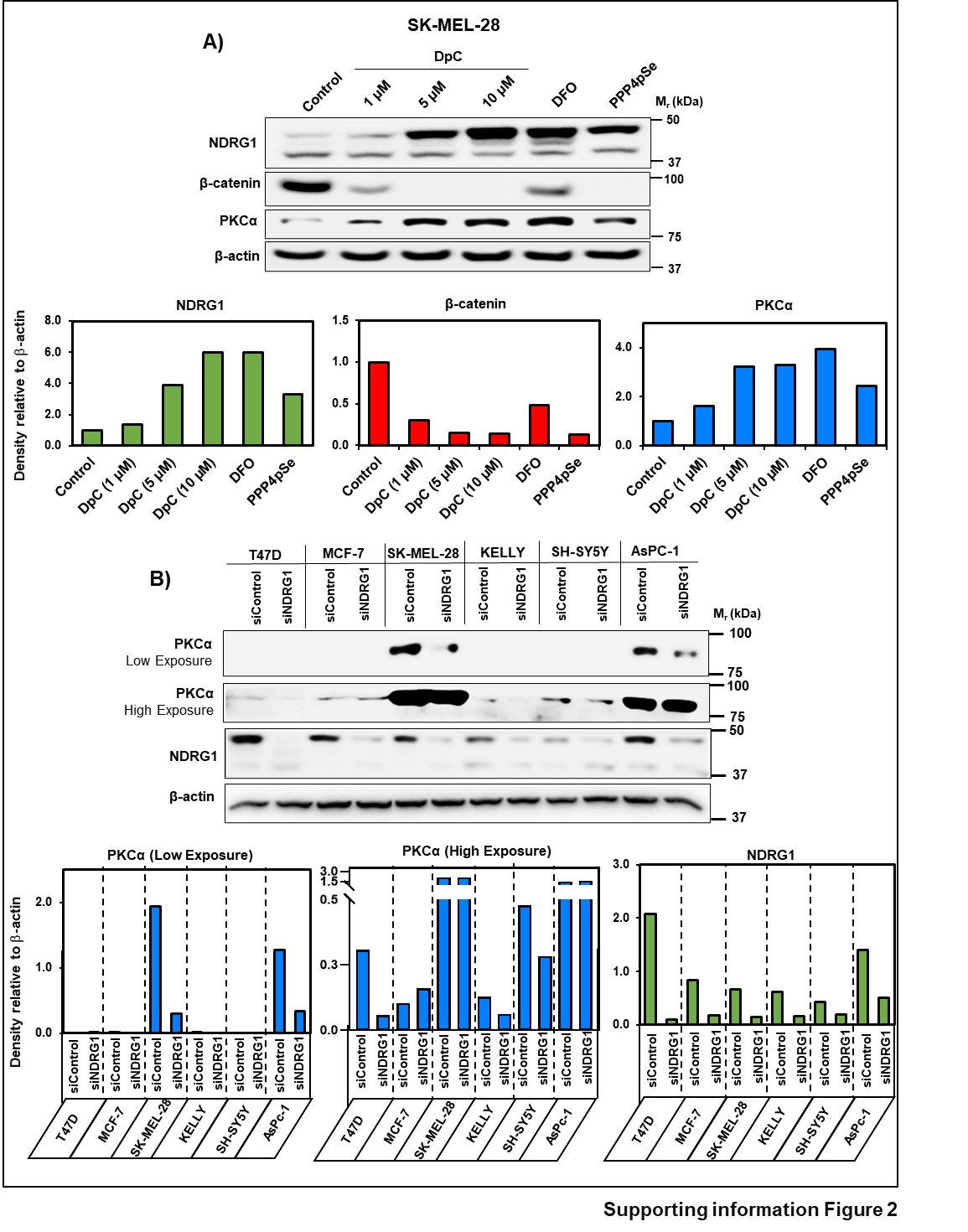
**

**Supporting information Table 1: List of the primary and secondary antibodies used to perform the studies.**

| No. | Name of antibody | Catalogue # | Supplier | Dilution |
| --- | --- | --- | --- | --- |
| 1. | NDRG1 Goat polyclonal antibody | ab37897 | Abcam Inc. | 1:5000 |
| 2. | PKCα (D7E6E) Rabbit mAb | 59754 | Cell Signaling Technology | 1:5000 |
| 3. | β-Catenin (6B3) Rabbit mAb | 9582 | Cell Signaling Technology | 1:5000 |
| 4. | Phospho-β-Catenin (Ser552) Rabbit mAb | 5651 | Cell Signaling Technology | 1:2000 |
| 5. | Non-phospho (Active) β-Catenin (Ser33/37/Thr41) Rabbit AntibodyT | 8814 | Cell Signaling Technology | 1:2000 |
| 6. | Phospho-β-Catenin (Ser33/37/Thr41) Rabbit Antibody | 9561 | Cell Signaling Technology | 1:2000 |
| 7. | Akt Rabbit Antibody | 9272 | Cell Signaling Technology | 1:5000 |
| 8. | Phospho-Akt (Ser473) (D9E) XP® Rabbit mAb | 4060 | Cell Signaling Technology | 1:1000 |
| 9. | PAK4 (G222) Rabbit Antibody | 62690 | Cell Signaling Technology | 1:2000 |
| 10. | GSK-3β (D5C5Z) XP® Rabbit mAb | 12456 | Cell Signaling Technology | 1:2000 |
| 11. | Phospho-GSK-3β (Ser9) (D85E12) XP® Rabbit mAb | 5558 | Cell Signaling Technology | 1:1000 |
| 12. | Phospho-GSK-3b (Tyr216) Polyclonal Rabbit Antibody | 602-190 | Thermo Fisher Scientific | 1:1000 |
| 13. | FRAT1 Rabbit antibody [EPR3900] | ab108405 | Abcam | 1:2000 |
| 14. | Cyclin D1 (92G2) Rabbit mAb | 2978 | Cell Signaling Technology | 1:1000 |
| 15. | Monoclonal Mouse β-Actin antibody | A5441 | Sigma-Aldrich | 1:10,000 |
| 16. | Goat IgG secondary | A5420 | Sigma-Aldrich | 1:10,000 |
| 17. | Mouse IgG secondary | A4416 | Sigma-Aldrich | 1:10,000 |
| 18. | Rabbit IgG secondary | A6154 | Sigma-Aldrich | 1:10,000 |
| 19. | Rabbit (DA1E) mAb IgG XP® Isotype Control | 3900 | Cell Signaling Technology | Final conc. depending IP antibody used in the pulldown process |
| 20. | Mouse (G3A1) mAb IgG1 Isotype Control | 5415 | Cell Signaling Technology | Final conc. depending IP antibody used in the pulldown process |
| 21. | Anti-rabbit IgG (H+L), F(ab')2 Fragment (Alexa Fluor® 555 Conjugate) | 4413 | Cell Signaling Technology | 1:1000 |
| 22. | Anti-mouse IgG (H+L), F(ab')2 Fragment (Alexa Fluor® 488 Conjugate) | 4408 | Cell Signaling Technology | 1:1000 |
| 23. | Anti-Goat IgG (H+L) Cross-Adsorbed Secondary Antibody, Alexa Fluor™ 647 | A-21447 | Thermo Fisher Scientific | 1:1000 |
| 24. | PKC alpha mouse antibody [133] | Ab11723 | Abcam | 1:1000 |
| 25. | Phospho-PKCα/β II (Thr638/641) Antibody | 9375S | Cell Signaling Technology | 1:1000 |
| 26. | NDRG1 Rabbit antibody [EPR5593] | Ab124689 | Abcam | 1:50 |
| 27. | β-Catenin antibody [BLR086G] | Ab265591 | Abcam | 1:50 |
| 28. | β-Catenin antibody | PA5-143193 | Thermo Fisher Scientific | 1:300 |
